# Chains of Spin-Crossover Complexes Synthesized on the Ag(111) Surface

**DOI:** 10.1021/jacs.6c15342

**Published:** 2026-09-25

**Authors:** Biswajit Pabi, Lydia Adam, Jan Krahmer, Beshr Madaraty, Cyrille Barreteau, Alexander Weismann, Felix Tuczek, Richard Berndt

**Affiliations:** † Institut für Experimentelle und Angewandte Physik, Christian-Albrechts-Universität zu Kiel 24098 Kiel, Germany; ‡ Institut für Anorganische Chemie, Christian-Albrechts-Universität zu Kiel 24098 Kiel, Germany; § Université Paris-Saclay, CEA, CNRS, SPEC 91191 Gif-sur-Yvette, France

## Abstract

Artificial spin chains composed of switchable units are a key objective in molecular spintronics, but their creation on surfaces is a challenge. Spin-crossover molecules allow for the reversible switching between spin states and could provide a means of creating such functional nanostructures through on-surface synthesis. We report the first on-surface thermally activated Ullmann coupling of spin-crossover complexes on a Ag(111) surface. Using scanning tunneling microscopy, we demonstrated the formation of molecular chains with electrically tunable spin states. The conductance spectra of individual molecules in chains exhibit spin-flip excitations. A comparison to a Heisenberg spin chain shows that the spins are antiferromagnetically coupled. Reversible manipulation of the molecules in the chains suppresses the spin excitations, indicating a transition from high spin to low spin *S* = 0. Overall, these observations confirm that Ullmann coupling on surfaces is a viable strategy for engineering electrically programmable spin chains with a spin-crossover functionality.

## Graphical abstract

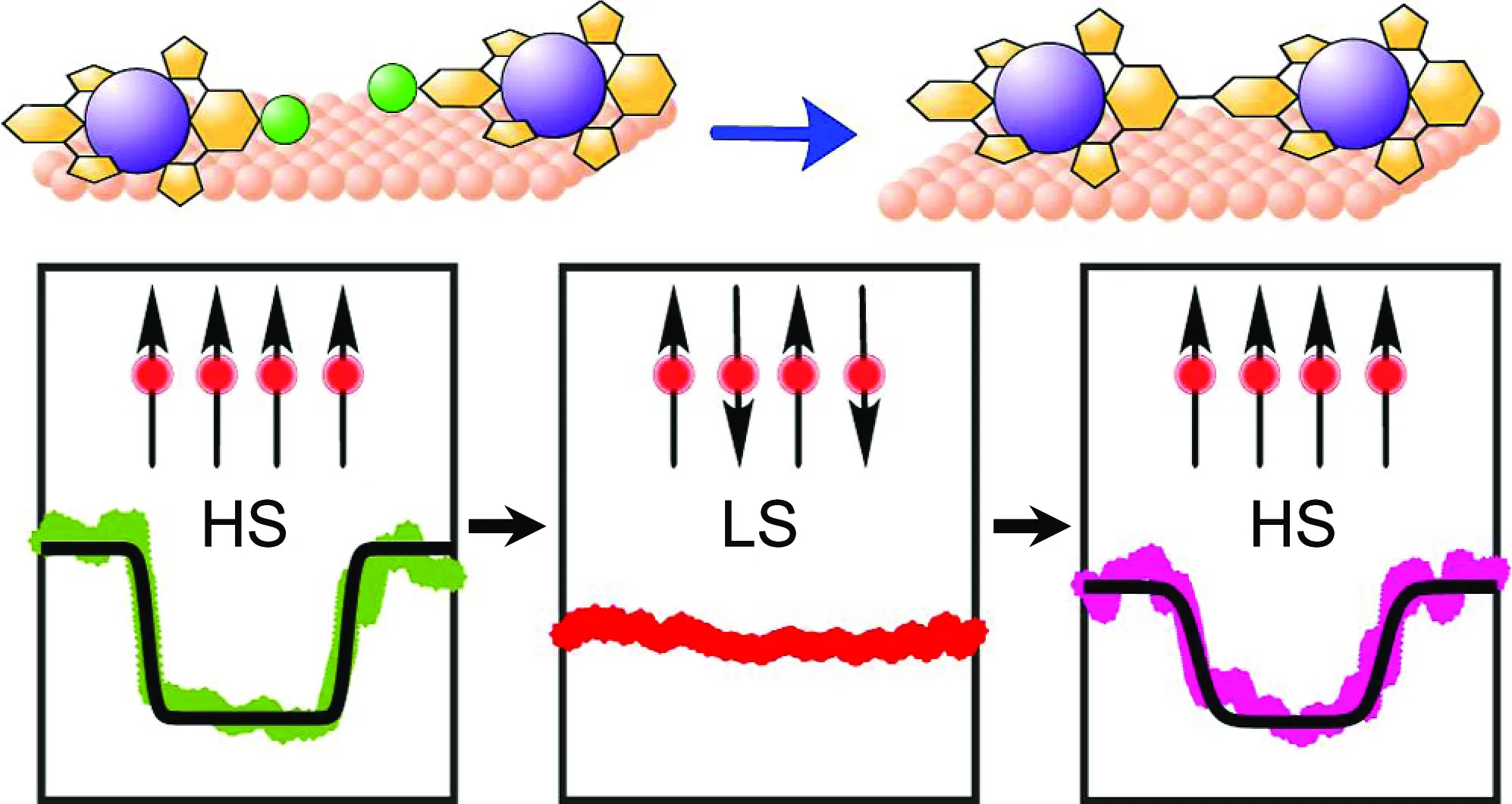

## Introduction

Spin chains offer attractive research opportunities that can lead to technological applications. Scanning tunneling microscopy (STM) has been used very successfully to construct and study atomic chains, as reviewed by Choi et al.^1^

Molecule-based spin chains are a more recent development. While the direct deposition of complexes with more than one spin-carrying center from the gas phase was successful in a few cases,^2–6^ the large size and the structural flexibility of such complexes, however, severely limit the scalability of this method. Therefore, chains or ribbons are typically prepared by inducing on-surface reactions between suitable precursor molecules.^7–9^ In particular, a wide variety of graphene-related structures have been obtained and studied.^10–19^ Spins are introduced in these structures by suitable topologies of the π-bond network or by including heteroatoms such as N.^16^ Chains and networks of planar organometallic complexes have also been investigated.^20–27^

The presence or absence of a spin in the structures discussed so far is directly related to the structure of the prepared array. Meanwhile, spin-crossover (SCO) complexes provide the opportunity to induce transitions between different spin states within a given molecule.^28–30^ This effect has also been observed at surfaces, where local excitation with electrons has, in some cases, provided control on the single-molecule level.^31–34^ Spin switching has also been achieved by adding or removing ligands from a spin-carrying center.^35–37^

It seems natural to apply the concept of on-surface synthesis to SCO complexes and to produce reconfigurable arrays of spin centers.^38,39^ However, SCO complexes typically comprise a transition metal ion surrounded by a three-dimensional ligand shell. The extent to which on-surface synthesis, which has mainly relied on planar precursor molecules so far, is applicable to three-dimensional SCO complexes is unknown, and challenges in synthesizing nonplanar nanographenes have been reported.^40^ However, some encouraging studies have used three-dimensional precursors to produce graphene-like ribbons^41^ and two-dimensional polymers.^42^ In addition, fullerenes and helicenes synthesized from planar precursors,^43–45^ a curved cycloarene,^46^ and a diamantane dimer^47^ have been reported. Chains of three-dimensional subunits were obtained by manipulation with local probes.^48^

Here, we report the coupling of the complex [Fe­(pzpypyr-Cl)_2_] to chains on a Ag(111) surface (Figure 1). The ligand pzpypyr is the anion of 4-Chloro-2-(3,5-dimethyl-1H-pyrazol-1-yl)-6-(1H-pyrrol-2-yl)-pyridine. Terminal Cl ions were implemented to enable an Ullmann coupling^49^ between adsorbed complexes. Using low-temperature STM, we found the adsorbed complexes to be spin-switchable. Control experiments with an analogous Zn complex corroborate this interpretation. Via suitable annealing, nearly one-dimensional arrays of complexes were obtained. The STM data suggest that these chains result from an on-surface reaction. Single subunits of the chains may be switched between different spin states, as evidenced through spectroscopy of spin excitations. While the present results leave room for improvement in terms of the homogeneity and ordering of the obtained supramolecules, they demonstrate that on-surface synthesis is not limited to planar structures. It is also feasible for a three-dimensional SCO complex and enables the preparation of configurable spin chains.

**Figure 1:**
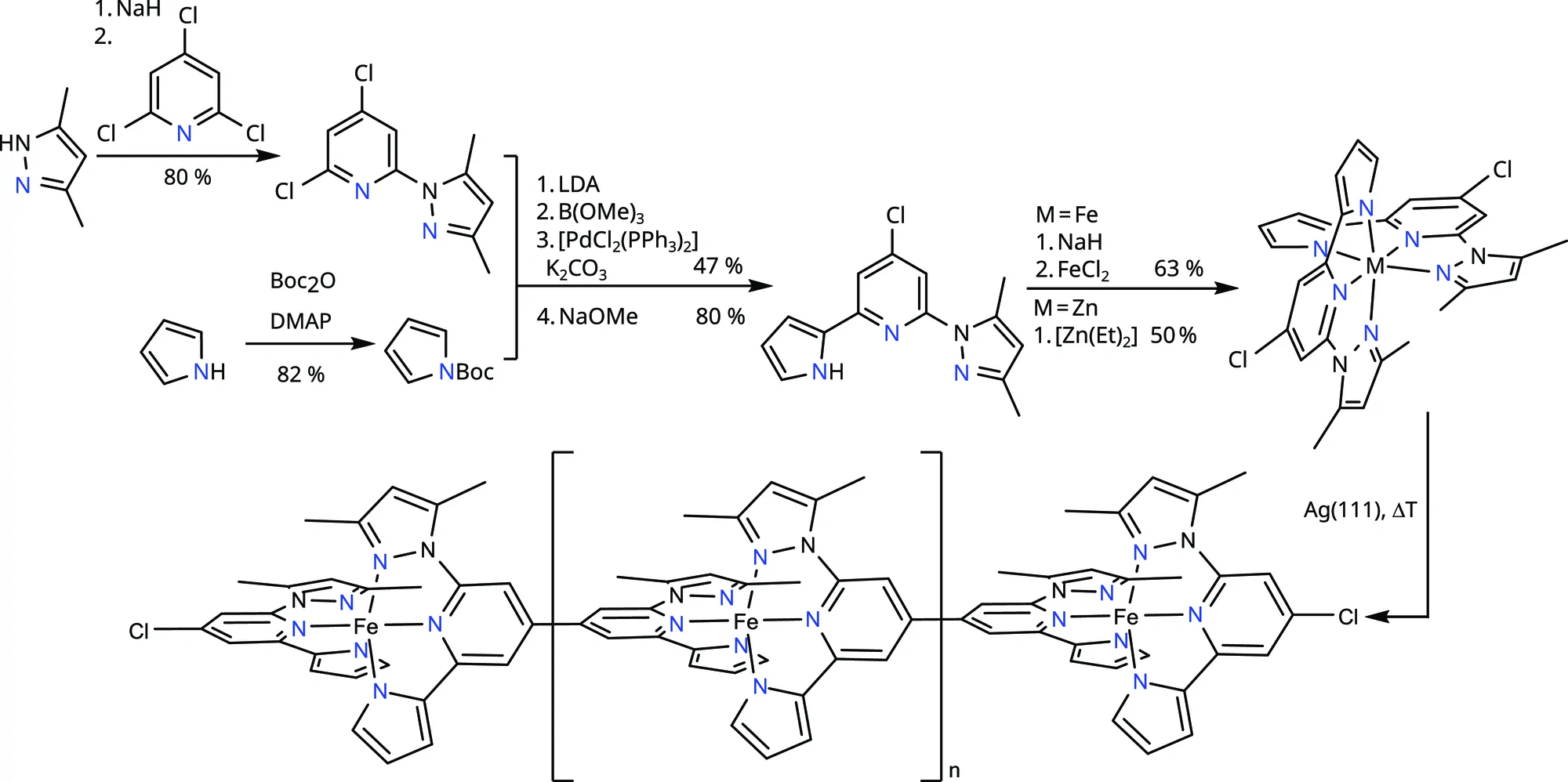
Synthesis routes. Top: Synthesis of [M­(pzpypyr-Cl)_2_], M = Fe and Zn. Bottom: Ullmann coupling to linear chains.

## Results and Discussion

### Synthesis and Characterization of [Fe­(pzpypyr-Cl)_2_]

The target complex must (i) be vacuum-evaporable, (ii) be spin-switchable on a metal surface, and (iii) enable Ullmann coupling. To create such systems, we focused on neutral, homoleptic Fe­(II) complexes supported by tridentate monoanionic ligands as contained in the complex [Fe­(pypypyr)_2_].^50^ The tridentate pypypyr ligand consists of a central pyridine unit featuring a deprotonable pyrrole at the 2-position and a charge-neutral pyridine at the 6-position. As a potential building block for the on-surface synthesis of a spin-crossover chain, we initially targeted the derived complex [Fe­(pypypyr-Cl)_2_] in which central pyridine rings of the pypypyr ligands are substituted by chlorine atoms in 4-position. Upon transition to the high-spin state, however, this complex was found to exhibit large bending of the tridentate ligands with respect to the axis through the central pyridine rings (φ = ∠(Pyridine-C_4_ – Fe–Pyridine-C_4_) decreases from 178.7° to 169.4°, Δφ = 9.3°). This may be detrimental for spin switching in the envisioned Ullmann-coupled linear chain. To minimize this distortion, we explored alternatives via density functional theory (DFT) calculations. In the optimized design, the charge-neutral pyridine is replaced with a 1,3-dimethylpyrazole unit while retaining the pyrrole moiety, leading to the ligand pzpypyr.

The synthesis of the Cl-substituted ligand pzpypyr-Cl and the derived complexes [M­(pzpypyr-Cl)_2_] (M = Fe and Zn) is depicted in Figure 1. 2,4,6-Tricholoropyridine was substituted with 1,3-dimethylpyrazole and subjected to Suzuki coupling with boc-protected pyrrole, followed by deprotection of the latter. The resulting ligand pzpypyr-Cl was reacted with FeCl_2_ and diethyl zinc to generate [Fe­(pzpypyr-Cl)_2_] and [Zn­(pzpypyr-Cl)_2_], respectively, adding NaH in the case of the iron complex to deprotonate the ligand. Details of the synthesis and analytical data are provided in the Supporting Information.

The single-crystal X-ray structure of the complexes can be found in the Supporting Information, Table S1. The [Fe­(pzpypyr-Cl)_2_] complex shows Fe–N bond lengths of 190 to 199 pm, corresponding to a low-spin configuration (Supporting Information, Table S1). Mössbauer spectra of [Fe­(pzpypyr-Cl)_2_] at 290 and 80 K exhibit parameters compatible with this (Supporting Information, Figure S1).^50–55^ Unfortunately, the corresponding high-spin structure is not available. However, it can often be approximated by that of the corresponding Zn­(II) complex, which in the case of [Zn­(pzpypyr-Cl)_2_] has Zn–N bond lengths between 209 and 229 pm. Importantly, the change of φ upon transition to the high-spin structure is much smaller (φ = 169.8° for the Fe­(II) and φ = 167.2° for the Zn­(II) complex, Δφ = 2.6°) in the pzpypyr than in the original pypypyr system (Δφ = 9.3°, see above.). Further structural details can be found in the Supporting Information.

### Adsorption on Ag(111) and Chain Formation

Figure 2a shows a STM topograph of surface area covered with submonolayer amounts of [Fe­(pzpypyr-Cl)_2_]. The image was obtained after deposition at ambient temperature and cooling to 4.5 K over a period of ≈1 h. The complexes seem to be randomly distributed over the substrate terrace, with some accumulation on the upper terrace near the step edge. The close-up view in panel (b) shows asymmetric structures (an example is marked by a white circle) that we attribute to monomers of [Fe­(pzpypyr-Cl)_2_] and small clusters, presumably dimers. From an analysis of large surface areas, we find that the monomer is the most prevalent feature and that the dimers arranged side-by-side (red circle) are observed more frequently than head-to-head dimers (orange circle).

**Figure 2:**
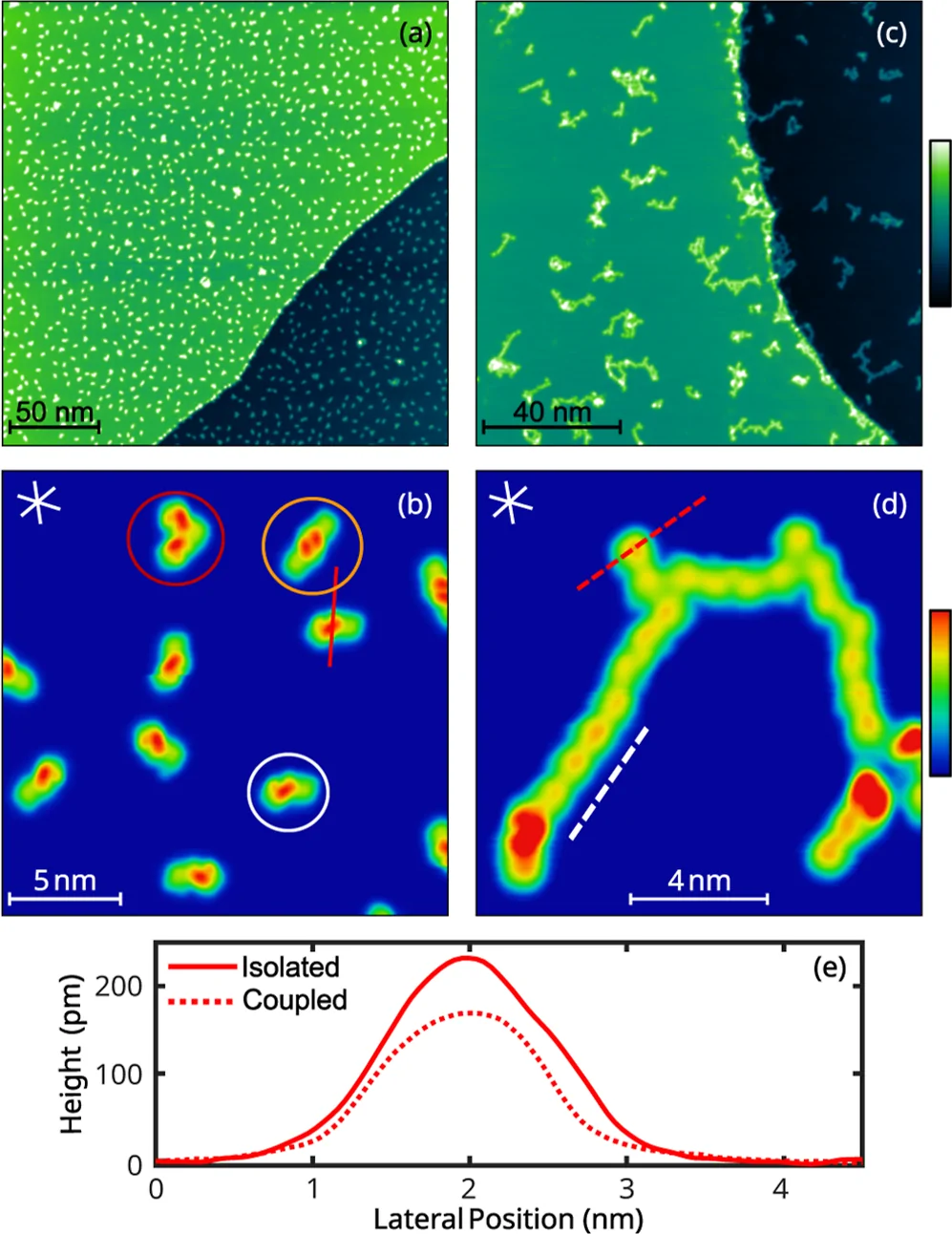
Constant-current topographs of a submonolayer coverage of [Fe­(pzpypyr-Cl)_2_] on Ag (111). (a) Overview of a (250 nm)^2^ area before annealing. *V* = – 0.2 V, *I* = 10 pA. (b) More detailed (20 nm)^2^ topograph from the sample in panel (a). (c) Overview image obtained after annealing. (130 nm)^2^. (d) Detailed topograph from the sample in panel (c). (13 nm)^2^. The overviews show two Ag terraces, separated by monatomic steps. The detailed images focus on a single terrace. White stars in (b,d) indicate the densely packed directions of the Ag(111) substrate. The white dashed line in (d) marks a straight section of a chain. The false colors in (a–d) cover height ranges of 485, 250, 490, and 205 pm, respectively. (e) Height profiles corresponding to the solid and dashed red lines in (b,d) through an isolated and a coupled molecule, respectively.

We performed DFT calculations of the isolated complex on Ag(111) in *S* = 0 (LS) and *S* = 2 (HS) states and simulated STM images of the optimized geometries (Figure 3). While the structural differences between the HS and LS states are reflected in the images, an unequivocal identification of the spin state from the experimental image (Figure 3e) is not possible. We neglect the small difference in the azimuthal orientations of the calculated HS and LS states because the total energy minimum found in the calculations of the HS state is very shallow with respect to the azimuthal angle.

**Figure 3:**
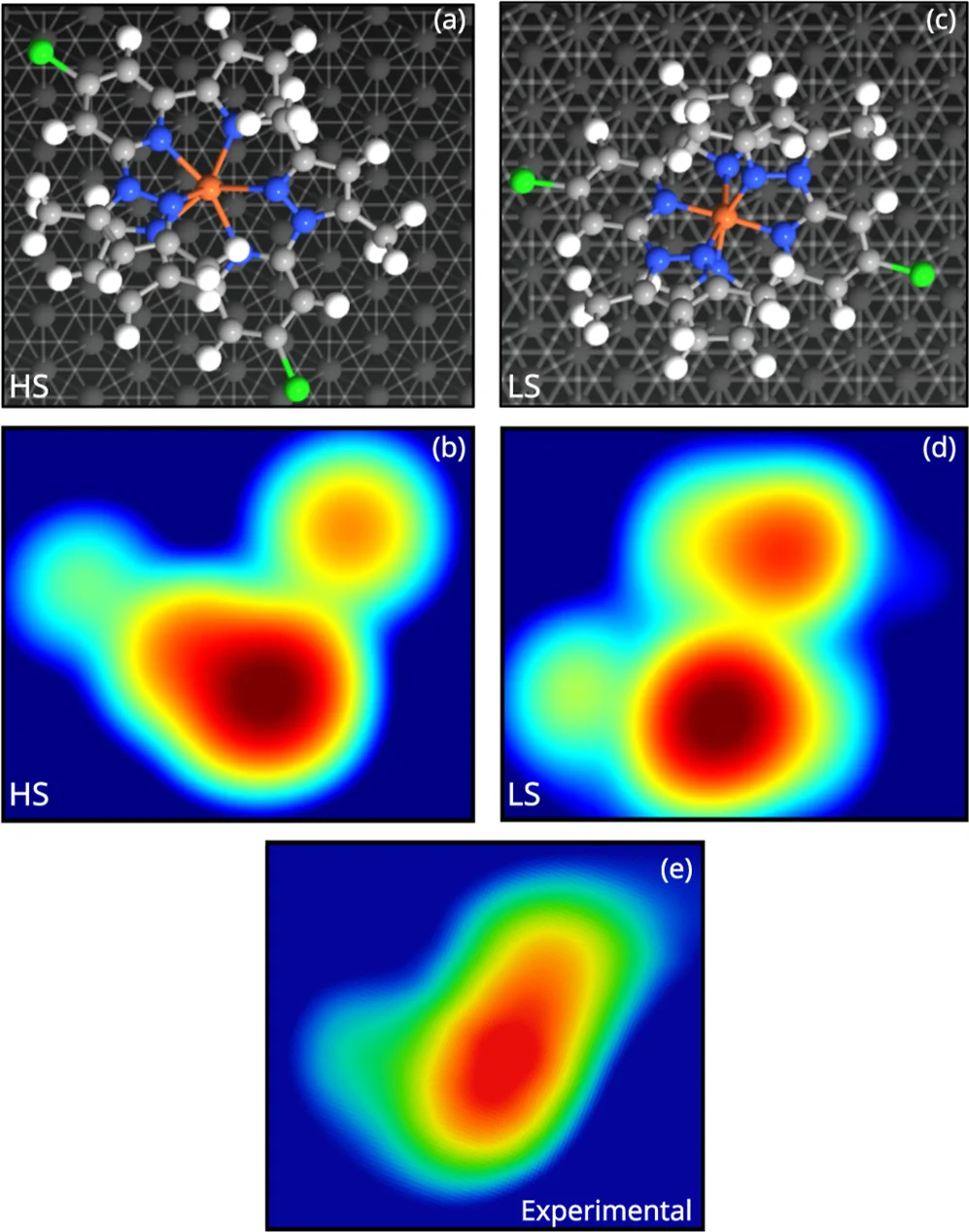
DFT results: (a,b) Optimized geometries of [Fe­(pzpypyr-Cl)_2_] on Ag(111) in the HS and LS states. (c,d) Corresponding simulated STM images for *V* = 0.3 V. (e) Experimental topograph of an isolated molecule, displayed on the same scale as the simulated images. The experimental image has been rotated to optimize the match with the simulated HS image.

Next, the sample was heated to 420 K for a period of 30 min and then cooled back to 4.5 K. After that, the arrangement of the complexes is drastically different. A small number of larger clusters is observed (Figure 2c), some of which are comprised of longish straight segments (Figure 2d). Typically, the straight chain segments are limited to a few molecules (example indicated by a white dashed line in panel d) and are oriented at an angle of ≈13^°^ with respect to the densely packed direction of the substrate. Further examples of chains are shown in the Supporting Information, Figure S3.

We argue that an Ullmann coupling occurred, resulting in bipyridine linkages between the complexes in these segments (Figure 1, bottom). The fact that isolated complexes appear 50 pm higher than the subunits in chains (Figure 2e) indicates that some change to the molecules occurred. More importantly, the separation between the subunits in chains is ≈ (1.06 ± 0.7) nm. This value is substantially smaller than the Cl–Cl distance of the original complex (1.329 nm, obtained via gas-phase DFT calculations). Intriguingly, it is also smaller than the estimated length of the dechlorinated subunit (1.13 nm, maximal C–C distance plus one bond length). However, our DFT calculations of a dimer in the gas phase and an isolated complex on the substrate show that opposite ligands are not perfectly aligned. Rather, the angle ∠(Pyridine-C_4_ – Fe – Pyridine-C_4_) is φ = 166° in the gas phase and 151° on the substrate, which effectively reduces the period of the chain. To obtain a reduction from 1.13 to 1.06 nm, an angle of ≈160° would be required. This value lies between the DFT results for the gas-phase and for the isolated adsorbed complex. The deviation can be attributed to surface–adsorbate anchoring and intermolecular forces that are neglected in the calculated models. Finally, the subunits in chains were much more stable toward imaging at elevated current than isolated complexes.

The chain networks exhibit arbitrary orientations, with perfectly straight domains limited to only 3 or 4 subunits. This structural inhomogeneity seems to be disturbing at first glance. However, this is a natural consequence of the bending of the complex. Depending on its orientation at the surface, the complex may be in one of two chiral states, denoted as R and L, which correspond to clockwise and counterclockwise deviations of one ligand from the axis connecting the other ligand and the Fe center. A chain starting with an R unit can propagate via the subsequent additions of R or L complexes. For a tetramer starting with R (Supporting Information, Figure S12), this branching yields 8 distinct conformers (additional 8 would emerge starting with L). Crucially, according to this model, a straight, periodic section is obtained only through alternating (R-L-R-L) units.

### STM-Induced Transitions

Figure 4 shows the current–voltage characteristics, *I*(*V*), of [Fe­(pzpypyr-Cl)_2_] complexes. The data cover several forward and backward sweeps over the voltage ranges shown. For isolated complexes (Figure 4a), bistability is observed at both voltage polarities, while the molecules in chains (Figure 4b) are clearly bistable only at negative sample voltage. Moreover, isolated complexes occasionally exhibit three distinct states (Figure S4). The data for isolated molecules were obtained from molecules at step edges because *I*(*V*) measurements of other molecules induced translational motion (Figure S5 of the Supporting Information).

**Figure 4:**
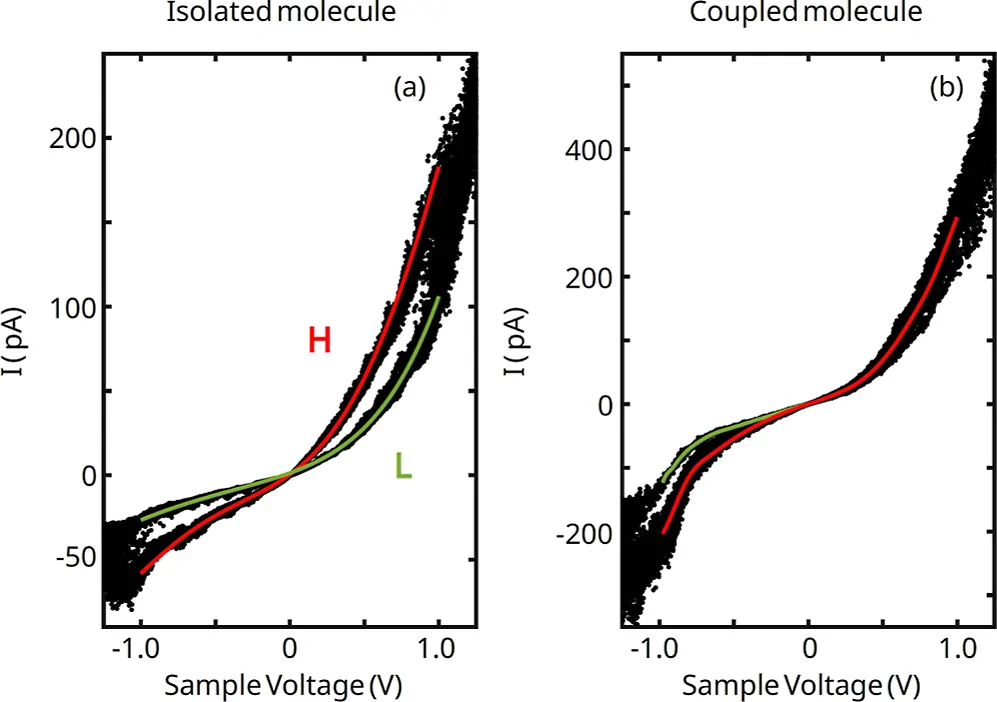
Bistability in *I*(*V*) characteristics. Panels (a,b) show *I*(*V*) data measured from an isolated complex and from a complex in a chain, respectively. The data points were obtained during 20 consecutive voltage sweeps from negative to positive voltages and back. Two distinct conductance states labeled L and H are observed for the isolated molecule when |*V*| exceeds 0.75 V. The transitions between L and H are abrupt on the time scale of the experiment. Red and green lines show mean values calculated by separately averaging high and low current branches. In contrast, the complex in a chain is bistable only at negative bias, *V* < – 0.85 V. Prior to voltage sweeps, the current feedback loop was opened at *V* = – 1.5 V and *I* = 50 pA (isolated) or 300 pA (in chain).

The isolated complexes as well as the subunits of chains could be reversibly modified by applying slightly elevated voltages. Figure 5a–c shows a manipulation sequence for an isolated complex. Starting from the pristine state P, a transition is induced to state T1 that exhibits slightly different image contrast. Further pulses induce a transition to state T2, which appears to be identical to the pristine state. Isolated molecules were fairly unstable and often moved laterally or rotated as shown in the Supporting Information, Figure S6. Molecules in chains (Figure 5d–f), however, usually did not suffer from these effects. Rather, a reduction of their apparent height by ≈ 40 pm was consistently observed (Supporting Information, Figure S7). This reduction was reversible through further voltage pulses, although the final height occasionally was different from the initial value. Induced transitions sometimes occurred not only in the complex right below the tip but also in adjacent molecules. From time series *I*(*t*), we found switching rates that are proportional to *I*, indicating a single-electron process. These rates correspond to yields of ∼10^–6^ transitions per electron.

**Figure 5:**
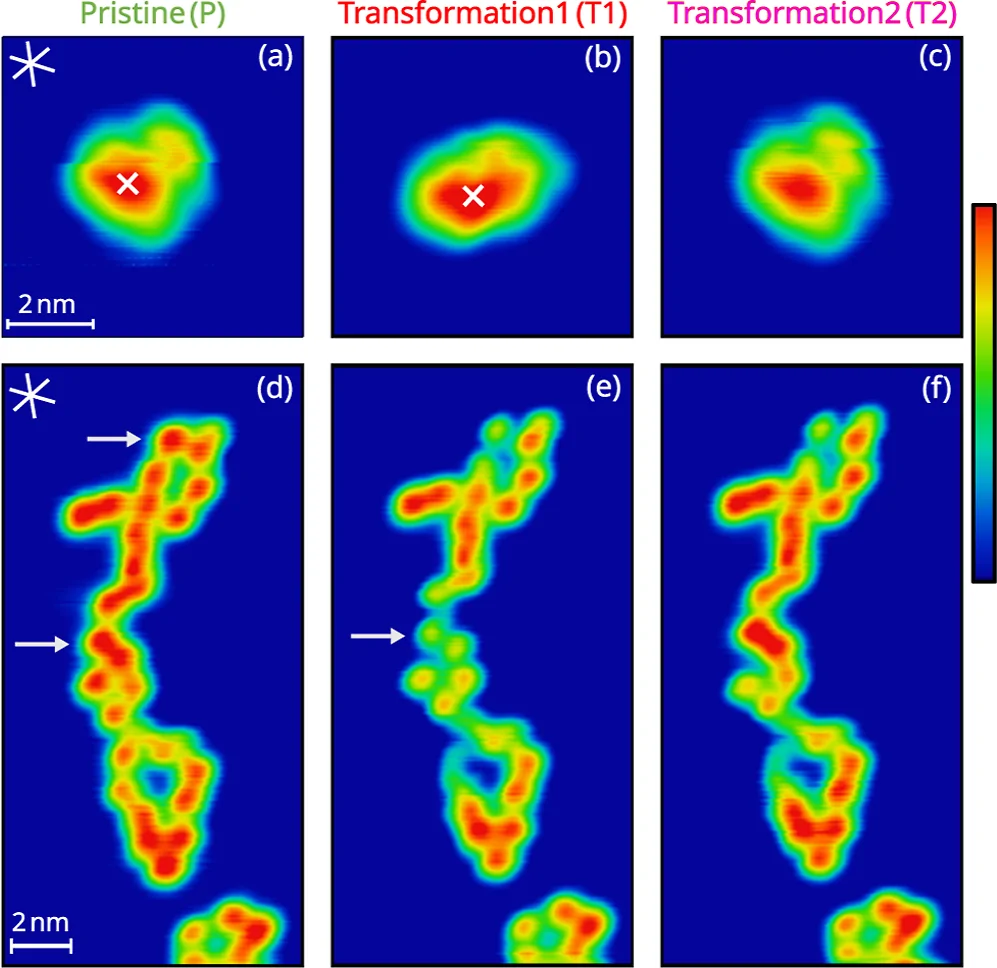
Reversible current-induced transitions. (a–c) Sequence of topographs (−0.4 V, 7 × 7 nm^2^) of an isolated [Fe­(pzpypyr-Cl)_2_] complex in its pristine state, after −1.3 V pulses at the position indicated by a cross, and after further pulses. Pulses of 0.5 s duration were applied with current feedback enabled (5 pA) until a change of the tip height was observed. (d–f) Sequence of topographs (10 × 20 nm^2^) illustrating the reversible transitions of some complexes in a molecular chain. 1.5 V pulses were used at the positions indicated by arrows. White lines indicate the densely packed directions of the substrate. The false colors span height ranges of 160 and 145 pm in the upper and lower row of images, respectively.

### Low-Energy Excitations

d*I*/d*V* spectra of the low-energy excitations are shown in Figure 6. The spectra of the isolated molecules in all states, pristine P, T1 after a first transition, and T2 after reverting the transition, are featureless (Figure 6a). In contrast, complexes in chains in the pristine and the reverted states exhibit clear step-like rises of the differential conductance by 60% or more at |*V*| = 9 meV (Figure 6b). Such features are typical of spin-flip excitations.^56–58^

**Figure 6:**
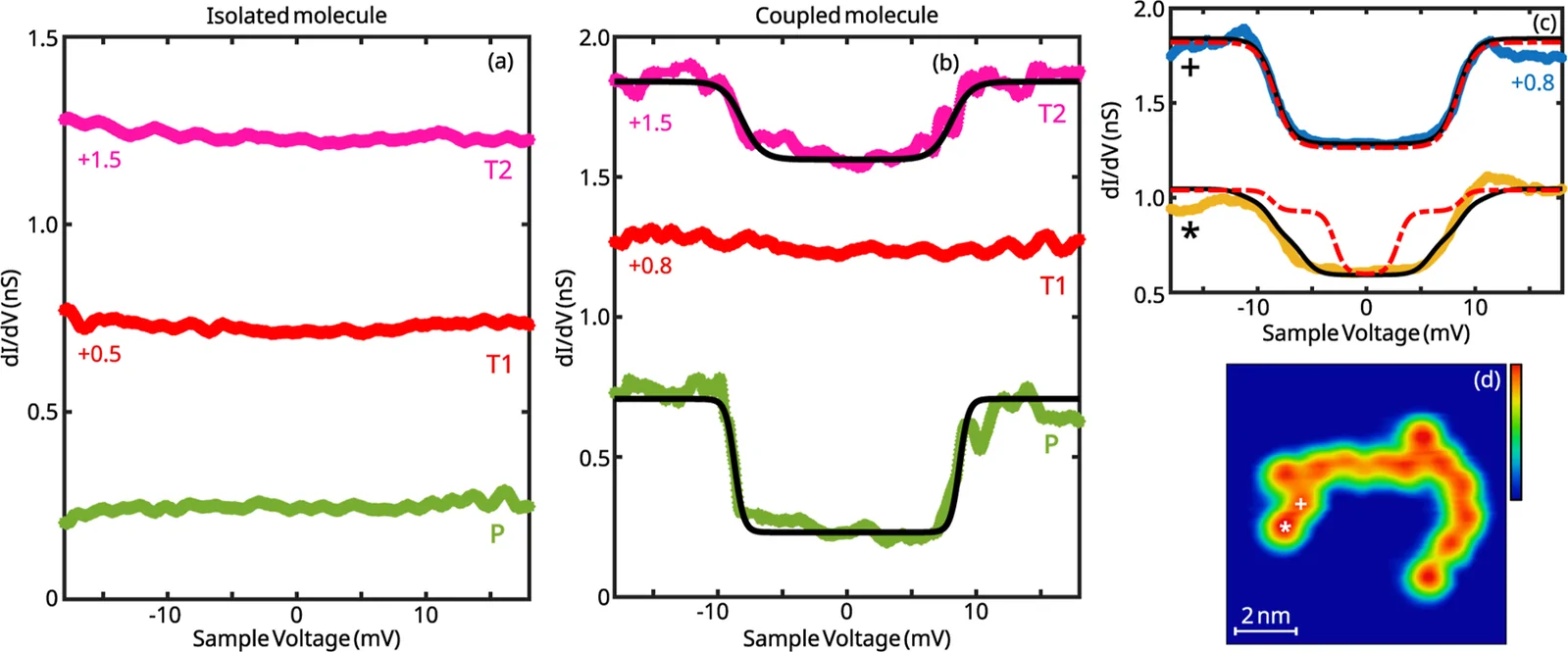
Low-bias excitations. (a) Differential conductance spectra (d*I*/d*V* vs sample voltage) measured above the highest point of an isolated molecule in before manipulation (P), after inducing a transition (T1), and after reversal of the transition (T2). (b) The same sequence of spectra obtained from a complex in a chain. Prior to the voltage sweeps, the current feedback loop was opened at *V* = – 20 mV and *I* = 200 pA. Corresponding topographs are shown in Figure 5. The chain spectra P and T2 were modeled (black lines) with the Hamiltonian of eq 1 using parameters *N* = 3, *S* = 2, and *J* = – 2.9 meV. (c) Differential conductance spectra (d*I*/d*V* vs sample voltage) acquired above the molecules indicated by * (at the edge) and + (at the interior) in the topograph. The data were measured at 4.2 K without an external magnetic field. For clarity, the upper spectrum is offset by 0.80 nS. Black solid and red dash-dotted lines show simulated spectra calculated using the Hamiltonian of eq 1 with parameters *N* = 3, *S* = 2, and *J* = – 2.9 (black) and 1.45 meV (red). (d) Constant-current topograph of a molecular chain on Ag(111) (9 × 9 nm^2^). The false colors cover a height range of 150 pm.

Spin excitations can originate from the single-ion anisotropy of individual molecules or, in coupled systems, from the exchange interaction between adjacent spins. Typically, the contribution from single-ion anisotropy manifests itself as a low-bias conductance step or dip.^58–61^ Conversely, magnetic exchange coupling in intermolecular architectures leads to excitations at higher energies.^6,25,62^ Our spectra usually do not resolve an effect of the single-ion anisotropy on the conductance, similar to previous reports on SCO complexes.^63^

Consequently, we neglect anisotropy and employ a Heisenberg exchange Hamiltonian, which was successfully used for atomic chains,^64^ to model the spin-excitation spectra

1 $$H=-J\sum_{i=1}^{N-1}\left(S_{x}^{i}S_{x}^{i+1}+S_{y}^{i}S_{y}^{i+1}+S_{z}^{i}S_{z}^{i+1}\right)$$

Here, *N* is the total number of spins in the chain, *S*_*x*_^*i*^, *S*_*y*_^*i*^, and *S*_*z*_^*i*^ are the Cartesian components of the spin operator at site *i*, and *J* is the exchange coupling between adjacent spins. Following ref 57, the spin-excitation spectra are expressed as a sum over temperature-broadened step-functions Θ­(*x*) at voltages *eV* = ± Δ_μν_:

2 $$\frac{dI^{i}}{dV}\left(V\right)=\sum_{\mu \nu }M_{\mu \nu }^{i}\left[\Theta \left(eV-\Delta_{\mu \nu }\right)+\Theta \left(-eV-\Delta_{\mu \nu }\right)\right]$$

The matrix element

3 $$M_{\mu \nu }^{i}=\frac{p_{\mu }}{\sum_{\mu }p_{\mu }}\sum_{\alpha =\left[x,y,z\right]}{\left|\left\langle \nu \left|S_{\alpha }^{i}\right|\mu \right\rangle \right|}^{2}$$

describes the probability of a spin-flip excitation at site *i* with energy Δ_νμ_ = *E*_ν_ – *E*_μ_ between the initial (final) eigenstates |μ⟩ (|ν⟩) of *H* having energies *E*_μ_ (*E*_ν_), with *p*_μ_ = exp­(−*E*_μ_/*k*_*B*_ *T*) being the Boltzmann factor. For a spin dimer (*N* = 2) with *S* = 2, the spin-excitation spectra can be reproduced equally well using ferromagnetic and antiferromagnetic *J*. The same applies to the center site *i* = 2 of a spin trimer (*N* = 3, see below), suggesting that the sign of *J* cannot be determined from these spectra alone.

Next, we utilize the fact that the excitation spectra of a Heisenberg spin chain can vary from site to site. Figure 6c shows the experimental spectra recorded on molecules at the edge (blue) and inside (yellow) the chain shown in Figure 6d. Although the spectral broadening differs slightly, the spectra are similar, in particular with respect to the excitation energies. Spectra exhibiting similar excitation energies, acquired on the other subunits of that chain, are provided in the Supporting Information, Figure S9. The spectrum of the interior molecule matches the calculated excitation spectrum of the central spin of a trimer, assuming either antiferromagnetic (AFM, *J* = – 2.9 meV) or ferromagnetic (FM, *J* = 1.40 meV) coupling (black solid and red dash-dotted lines, respectively). In contrast, the spectrum of the edge molecule is only reproduced when AFM coupling is used (black solid line). FM coupling leads to an excitation step at significantly lower energies (≈2.5 meV) and does not match the experimental observation. The spectra therefore indicate antiferromagnetic coupling, which is consistent with the expected AFM exchange interaction mediated by the bipyridine linker.^65^

We conclude that the spin excitations observed in the chains originate predominantly from intermolecular exchange. This mechanism naturally explains the absence of such features in the uncoupled monomers. The spectroscopic data demonstrate that the complexes in the chains initially are in a high-spin state. After the transition T1, spin excitations are no longer resolved, and the complexes most likely are in a low-spin state.

Occasionally, a complex was picked up by the tip during spectroscopic measurements, as confirmed by subsequent STM imaging. Spectra recorded with such a tip (Figure S10) do no longer exhibit the excitation near 10 mV as expected, because the exchange coupling to other complexes is weak. Rather, a low-energy excitation at ≈2.5 mV is resolved. As shown in the Supporting Information, this altered spectrum can be fit using only the single-ion anisotropy terms without invoking exchange coupling (Figure S10).

### Control Experiments: [Zn­(pzpypyr-Cl)_2_]

A series of control experiments were performed using [Zn­(pzpypyr-Cl)_2_], for which, owing to the complete filling of the Zn *d* shell, no SCO is expected. We found isolated complexes after deposition and aggregates after annealing (Supporting Information, Figure S11a,b). Neither the isolated complexes nor those in arrays showed bistability in *I*(*V*) data (Supporting Information and Figure S11c,d), and their d*I*/d*V* spectra did not reveal any spin excitations. These results confirm that the observed transitions of the current and the topographs of the Fe-based complex reflect a change in its spin state.

### Conclusions

We demonstrated that the chlorinated three-dimensional SCO complex [Fe­(pzpypyr-Cl)_2_] undergoes a thermally activated Ullmann reaction on the Ag(111) surface. This leads to the formation of chains of complexes that may be electrically switched between high- and low-spin states. Spectroscopy of the spin-flip excitations of the high-spin complexes reveals antiferromagnetic coupling along the chains with an exchange energy of *J* = – 2.9 meV. Our results demonstrate that Ullmann coupling on the surface is a viable strategy to engineer electrically programmable spin chains with intact spin-crossover functionality. The present chains exhibit a degree of disorder that can be attributed to the geometry of the subunits. Future research will focus on achieving more homogeneous chain formation by designing new compounds.

## Supplementary Material



## Notes

Synthesis procedures, materials characterization, experimental protocols, modeling, and additional STM and spectroscopy data (PDF)
