# Supplementary material for: Chains of Spin-Crossover Complexes Synthesized on the Ag(111) Surface
Source: J Am Chem Soc. 2026 Sep 25;148(39):42668–75. doi: 10.1021/jacs.6c15342 (PMC13651523; doi:10.1021/jacs.6c15342)
Supplement: Supplementary file 1 [file ja6c15342_si_001.pdf]

Chains of Spin-Crossover Complexes Synthesized on the Ag(111) Surface

Biswajit Pabi,† Lydia Adam,‡ Jan Krahmer,‡ Beshr Madaraty,‡ Cyrille Barreteau,¶ Alexander Weismann,† Felix Tuczek,*,‡ and Richard Berndt*,†

†*Institut für Experimentelle und Angewandte Physik, Christian-Albrechts-Universität zu Kiel, 24098 Kiel, Germany*

‡*Institut für Anorganische Chemie, Christian-Albrechts-Universität zu Kiel, 24098 Kiel, Germany*

¶*Université Paris-Saclay, CEA, CNRS, SPEC, 91191 Gif-sur-Yvette, France*

E-mail: ftuczek@ac.uni-kiel.de; berndt@physik.uni-kiel.de

1. Methods

1.1. STM Experiments

The Ag(111) substrate was prepared by repeated cycles of argon ion (Ar^+) sputtering and annealing at temperatures up to 800 K. STM tips were made from W wire by electrochemical etching and sputtering in ultrahigh vacuum (UHV). The complexes were deposited from a Knudsen cell onto the Ag(111) substrate held at room temperature. The experiments were performed in UHV using a custom-built STM operated at 4.5 K. $\mathrm{d}I/\mathrm{d}V$ spectra were measured by freezing the position of the STM tip and using a lock-in amplifier with a sinusoidal modulation (frequency: 833 Hz, amplitude: 2 mV) superimposed on the sample voltage and smoothed using a Savitzky-Golay filter. Constant current topographs were obtained with a sample voltage $V = -0.5\,\mathrm{V}$ at a current $I = 5\,\mathrm{pA}$ unless otherwise noted.

1.2. Computational Details

DFT modeling was performed with QuantumATK 2025.06[1] using norm conserving pseudopotentials with a medium basis set and PBE functional.[2] The DFT+U formalism was employed as well Grimme DFT-D3 dispersion corrections.[3] DFT+U is necessary to describe the energy balance between LS ($S = 0$) and HS ($S = 2$) states $\Delta E = E_{\mathrm{LS}} - E_{\mathrm{HS}}$ while Grimme DFT-D3 is essential to reproduce the molecule-substrate interaction. The U value has been set to 1.7 eV as in previous study for similar iron spin-crossover complexes. Using this value, the LS state is favored by $\approx -0.45\,\mathrm{eV}$ for the isolated complex. The Fe-N distances are in the ranges of $[190, 195]$ and $[210, 220]$ pm for the LS and HS states respectively.

The Ag(111) substrate was modeled with three atomic layers in an orthorhombic unit cell of dimensions 1.73, 1.5, and 2.5 nm along the x, y, and z axes, respectively. The x-axis is aligned with a nearest-neighbor direction of Ag(111), while the z-axis is normal to the surface. The slab unit-cell contains 108 silver atoms and 63 atoms of the molecule.

The geometry optimization was performed with a 2×2 grid of k points until forces were

below 0.01 eV/Å. Total energies and electron densities were calculated with a 4×4 grid. The geometry of the adsorbed molecule is identified via the orientation of the chlorine-iron-chlorine axis (Cl-Fe-Cl) relative to the Ag(111) surface.

2. Syntheses

The syntheses were performed under a nitrogen atmosphere unless mentioned otherwise using standard Schlenk techniques. A M. Braun Labmaster 130 glovebox was utilized for operations under an argon atmosphere. Commercial solvents and starting materials were used as received except solvents, marked with abs., which were dried and purified by distillation under an inert atmosphere using the following drying agents: Et_2O over $LiAlH_4$ (under Ar), DCM over CaH_2 (under N_2), and THF over potassium (under N_2). All solvents were destilled prior to use.

Flash column chromatography was performed using a Biotage Isolera One Flash Chromatography System with prepacked columns of the type CHROMABOND Flash BT25 SiOH. All solid crude products were dry-loaded by adding silica prior to removing the solvent in vacuo. NMR spectra were recorded at 298 K on a Bruker Avance 400 Pulse Fourier Transform spectrometer in deuterated solvents operating at a ^1H frequency of 400.1 MHz and ^{13}C frequency of 100.6 MHz. All NMR spectra were referenced to the residual protonated solvent signal (^1H: $\delta(CHCl_3) = 7.26$ ppm; δ $(CH_2Cl_2) = 5.32$ ppm), the solvent signal (^{13}C: δ $(CDCl_3)$ $= 77.16$ ppm; δ $(CH_2Cl_2) = 54.00$ ppm). With the help of DEPT-135 and two-dimensional correlation spectra (^1H,^1H-COSY, ^1H,^{13}C-HSQC, ^1H,^{13}C-HMBC) signals were assigned. Signal multiplicities are abbreviated as s (singlet), d (doublet), t (triplet), m (multiplet) and br. (broad signal).

Infrared spectra were recorded with neat samples on a Bruker Alpha FT-IR Spectrometer with a Platinum ATR module in a range of 4000 to 400 cm^{-1} with a resolution of 1.5 cm^{-1}. Signal intensities are marked as s (strong), m (medium), w (weak), and br (broad).

Elemental analyses were measured using a Euro Vector CHNS-O element analyser (Euro EA 3000) from HEKAtech by burning the samples in an oxygen stream in a tin pot. High resolution ESI mass spectra were recorded on a ThermoFisher Orbitrap spectrometer.

2.1. 2,4-dichloro-6-(3,5-dimethyl-1*H*-pyrazol-1-yl)pyridine

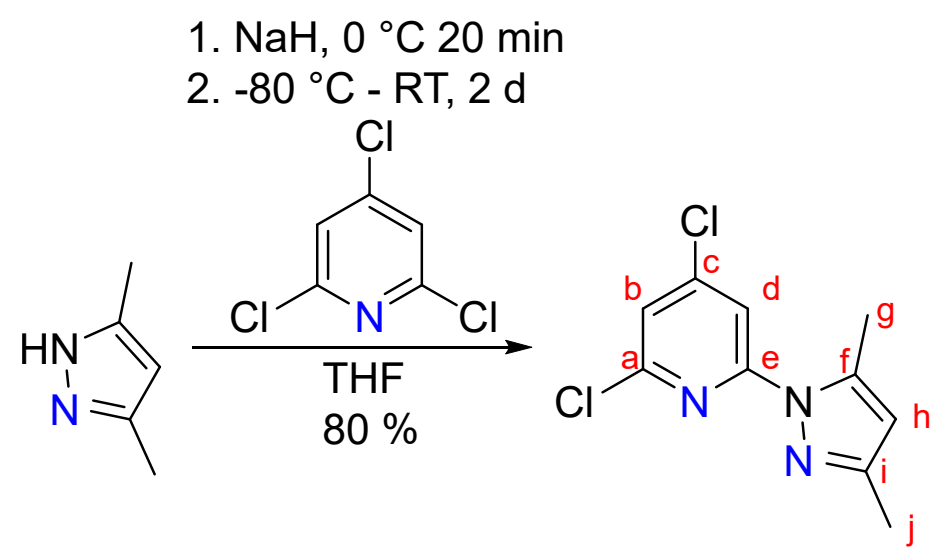

To our knowledge this kind of reaction is not established with trihalogenopyridines. For dihalogenopyridine the reaction mixture often has to be heated in order to achieve monosubstitution.[4,5] We found that monosubstitution at trihalogenopyridines is best achieved by starting the reaction at −80°C and then allowing for long reaction times at room temperature. At first 3,5-dimethylpyrazole (3.48 g, 36.2 mmol, 1.1 eq) was dissolved in 20 ml abs. THF and cooled to −80°C in a cold bath with ethanol and liquid nitrogen. Then 1.62 g (60 % dispersion in mineral oil, 39.5 mmol, 1.2 eq) of NaH were added in small portions in counterflow. The suspension was stirred and allowed to warm to room temperature over 5 hours, during which the mixture turned yellow. The mixture was cooled to −80°C again and 6.00 g (32.6 mmol, 1 eq) of trichlorpyridine was added in 5 ml abs. THF. The mixture was stirred and the temperature was kept at −80°C for 2 hours before it was allowed to warm to room temperature and stirred for 2 days. The mixture was washed two times with 60 ml deion. H_2O, dried over Na_2SO_4 and filtered. The product was purified by flash column chromatography (silica, cyclohexane + dichloromethane 0 % → 44 %) and obtained as a white solid with a yield of 6.65 g (26.21 mmol, 80 %).

^{1}H-NMR (400 MHz, $CDCl_3$): $\delta = 7.89$ (d, $^4J = 1.5$ Hz, 1 H, H_d), 7.14 (d, $^4J = 1.5$ Hz, 1 H, H_b, 6.0 (s, 1 H, H_h, 2.65 (d, $^4J = 0.9$ Hz, 3 H, H_g, 2.28 (s, 3 H, H_j ppm.

^{13}C-NMR (101 MHz, CDCl$_3$): δ = 153 (s, C_a), 151 (s, C_i), 149 (s, C_e), 147 (s, C_c), 143 (s, C_f), 120 (d, C_b), 113 (d, C_d), 110 (d, C_h), 15.1 (q, C_g), 13.8 (q, C_j) ppm.

IR (ATR): $\tilde{\nu}$ = 3110 (w), 3078 (w), 2991 (w), 2926 (w), 1571 (s), 1548 (s), 1481 (m), 1464 (m), 1432 (s), 1410 (s), 1392 (s), 1378 (s), 1335 (s), 1277 (s), 1257 (m), 1176 (m), 1141 (s), 1119 (m), 1092 (s), 1024 (m), 963 (s), 864 (m), 837 (m), 825 (s), 804 (s), 791 (s), 734 (s), 658 (w), 604 (m), 585 (w), 556 (m), 549 (m), 438 (m), 417 (m), 367 (w), 356 (w), 271 (w), 202 (m), 170 (m), 110 (m) cm^{-1}.

FT-RAMAN (KBr): $\tilde{\nu}$ = 3108 (w), 3079 (w), 2990 (w), 2977 (w), 2932 (m), 1572 (s), 1484 (m), 1438 (m), 1418 (m), 1392 (m), 1335 (w), 1281 (w), 1030 (w), 986 (s), 967 (m), 737 (m), 608 (w), 589 (m), 554 (w), 432 (w), 357 (m), 273 (w), 184 (m), 111 (m) cm^{-1}.

HR-ESI-MS (+): m/z = 242.02416 (calc. 242.02463 [M+H]$^+$).

Anal. Calcd for $C_{10}H_9N_3Cl_2$: C, 49.61 %; H, 3.75 %; N, 17.36 %. Found: C, 49.64 %; H, 4.00 %; N, 17.11 %.

2.2. *N-tert*-butyloxycarbonyl-pyrrole

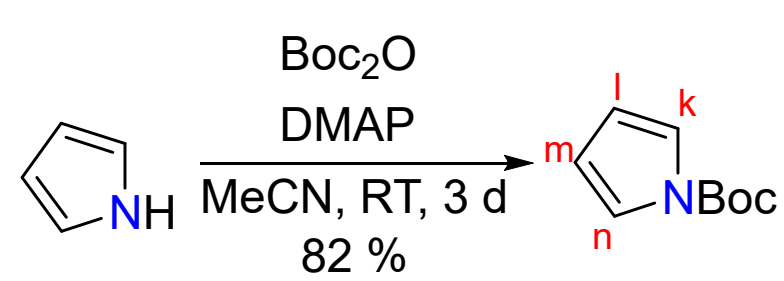

This compound was prepared after a procedure from the literature, but the purification was altered.[6] The pyrrole (4.31 g, 64,2 mmol, 1 eq) was freshly distilled (80 °C, 48 mbar) prior to use. It was then degassed via the freeze pump thaw method. In another flask DMAP (1.57 g, 12.9 mmol, 0.2 eq) and Boc$_2$O (16.8 g, 77.1 mmol, 1.2 eq) were dissolved in 40 ml abs. acetonitrile. To this solution the degassed pyrrole was added in 5 ml of abs. acetonitrile and rinsed with another 5 ml abs. acetonitrile. The solution was stirred in the dark at room temperature for 3 d. The solvent was removed *in vacuo*. The crude oily product was purified by flash column chromatography (silica, cyclohexane) and obtained as a colorless liquid in a yield of 8.77 g (52.5 mmol, 82 %).

^{1}H-NMR (400 MHz, DMSO-d$_6$): δ = 7.24 â€“ 7.22 (m, 2 H, $H_{\mathrm{k,\,n}}$), 6.25 (dd, ^3J = 2.61, 2.10 Hz, 2 H, $H_{\mathrm{l,\,m}}$), 1.55 (s, 9 H, H-Boc) ppm.

IR (ATR): $\tilde{\nu}$ = 3158 (w), 3115 (w), 2977 (m), 2934 (w), 1742 (s), 1585 (w), 1538 (w), 1469 (m), 1393 (m), 1368 (m), 1341(s), 1310 (s), 1250 (m), 1213 (w), 1197 (m), 1150 (s), 1072 (s), 1030 (m), 949 (s), 850 (m), 821 (m), 771 (m), 732 (s), 588 (m), 537 (m), 489 (m), 460 (m), 413 (m) cm^{-1}.

FT-RAMAN (KBr): $\tilde{\nu}$ = 3153 (w), 3117 (w), 2986 (m), 2933 (s), 1739 (m), 1467 (s), 1446 (m), 1399 (m), 1346 (m), 1310 (m), 1085 (m), 1037 (m), 953 (m), 922 (w), 855 (w), 831 (w), 757 (s), 492 (w), 314 (w), 173 (w) cm^{-1}.

2.3. *Tert*-butyl-2-(4-chloro-6-(3,5-dimethyl-1*H*-pyrazol-1-yl-)pyridin-2-yl)-1*H*-pyrrol-1-tert-butyloxycarbonyl

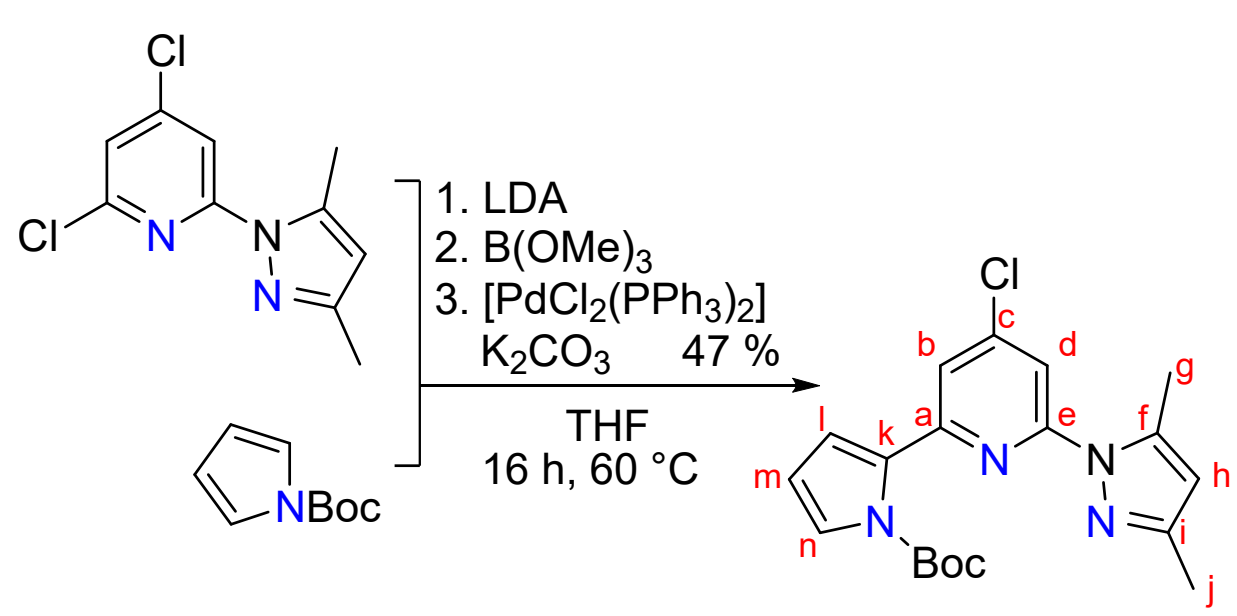

The protocol for this reaction was adapted from the literature.[7]

Firstly, LDA was freshly synthesized through dropwise addition of *n*-BuLi (2. 5 M in *n*-hexane, 12.83 ml. 32.1 mmol, 1.4 eq) to 3.24 g (4.51 ml, 32.1 mmol, 1.4 eq) diisopropylamine in 30 ml abs. THF at 0 °C. This mixture was stirred for 60 min after which it was cooled to -95 °C. The boc protected pyrrole (4.98 g, 29.77 mmol, 1.3 eq) was degassed via the freeze pump thaw method, dissolved in 30 ml abs. THF and added dropwise to the LDA solution. The mixture was stirred at -95 °C for 60 min before it was quenched with 4.52 g (4,9 ml, 43.5 mmol, 1.90 eq) trimethyl borate, allowed to warm to 0 °C and stirred for another 30 min. Under air 5.54 g (22.9 mmol, 1 eq) 2,4-dichloro-6-(3,5-dimethyl-1*H*-pyrazol-1-

yl)pyridine, 1.29 g (1.83 mmol, 0.08 eq) [Pd(PPh_3)$_2$$Cl_2$ in 50 ml THF and 6.33 g (45.8 mmol, 2 eq) K_2CO_3 in 30 ml deion. H_2O were added. The mixture was stirred at 60 °C for 16 h. The resulting brown solution with a white precipitate was extracted three times with 70 ml DCM, dried over Na_2SO_4, filtered and purified via flash column chromatography (silica, cyclohexane + ethyl acetate 0 % → 4 %) and obtained as a colorless oil with a yield of 4.03 g (10.81 mmol, 47 %).

^{1}H-NMR (400 MHz, CDCl$_3$): δ = 7.78 (d, ^4J = 1.7 Hz, 1 H, H_d), 7.30 (dd, ^3J = 3.4 Hz,^4J = 1.7 Hz, 1 H, H_n), 7.15 (d, ^4J = 1.7 Hz, 1 H, H_b), 6.43 (dd, ^3J = 3.4 Hz,^4J = 1.7 Hz, 1 H, H_l), 6.19 (t, ^3J = 3.3 Hz, 1 H, H_m), 5.98 - 5.96 (m, 1 H, H_h), 2.54 (d,^4J = 0.8 Hz, 3 H, H_g), 2.23 (s, 3 H, H_j), 1.31 (s, 9 H, H_{Boc}) ppm.

^{13}C-NMR (101 MHz, CDCl$_3$): δ = 153 (s, C_e), 151 (s, C_a), 150 (s, C_i), 149 (s, $C_{C=O}$), 145 (s, C_c), 142 (s, C_f), 133 (s, C_k), 125 (d, C_n), 120 (d, C_b), 117 (d, C_l), 113 (d, C_d), 110 (d, C_h), 84 (s, $C_{Boc\text{-}quart.}$), 27.7 (q, $C_{Boc\text{-}Me}$), 15.1 (q, C_g), 13.7 (q, C_j) ppm.

IR (ATR): $\tilde{\nu}$ = 3103 (w), 2980 (w), 2930 (w), 1744 (s), 1565 (s), 1475 (m), 1419 (s),1370 (m), 1307 (s), 1255 (m), 1147 (s) 1095 (m), 1070 (m), 1051 (m), 1028 (m), 1003 (m), 983 (m), 971 (m), 850 (s), 812 (m), 785 (s), 771 (m), 769 (m), 729 (s) cm^{-1}.

FT-RAMAN (KBr): $\tilde{\nu}$ = 3108 (w), 3079 (w), 2990 (w), 2977 (w), 2932 (m), 1572 (s), 1484 (m), 1438 (m), 1418 (m), 1392 (m), 1335 (w), 1281 (w), 1030 (w), 986 (s), 967 (m), 737 (m), 608 (w), 589 (m), 554 (w), 432 (w), 357 (m), 273 (w), 184 (m), 111 (m) cm^{-1}.

HR-ESI-MS (+): m/z = 373.14154 (calc. 373.14258).

2.4. 4-chloro-2-(3,5-dimethyl-1H-pyrazol-1-yl)-6-(1H-pyrrol-2-yl) pyridine (pzpypyr-Cl)

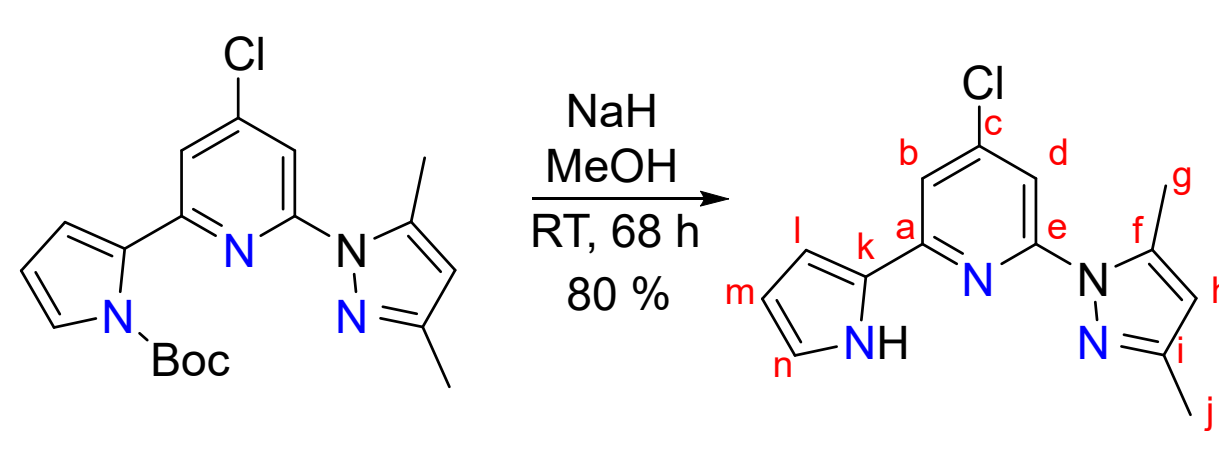

This reaction was performed similar to a protocol from the literature.[7]

In one flask 257 mg (60 % dispersion in mineral oil, 6.44 mmol, 0.6 eq) of NaH was added in small portions in N_2 counterflow to 15 ml of abs. methanol. In a separate flask the boc-protected ligand was dissolved in 20 ml of abs. methanol, added to the first flask and rinsed with another 5 ml of abs. methanol. The mixture was stirred at room temperature for 68 h before 2 M HCl was added to set the pH to 8. Solvents were removed *in vacuo* before the product was purified via flash column chromatography (silica, cyclohexane + ethyl acetate 0 % → 9 %) and obtained as a colorless solid with a yield of 2.33 g (10.73 mmol, 80 %).

^{1}H-NMR (400 MHz, CD_2Cl_2): δ = 9.20 (s, 1 H, NH), 7.58 (d, ^4J = 1.7 Hz, 1 H, H_d), 7.29 (dd, ^4J = 1.7 Hz, 1 H, H_b), 6.87 (td, ^3J = 2.7 Hz,^4J = 1.4 Hz, 1 H, H_n), 6.69 (ddd, ^3J = 3.8 Hz,^4J = 2.5, 1.4 Hz, 1 H, H_l), 6.23 (dt, ^3J = 3.7, 2.6 Hz, 1 H, H_m), 6.07 - 6.02 (m, 1 H, H_h), 2.64 (d,^4J = 0.9 Hz, 3 H, H_g), 2.27 (s, 3 H, H_j) ppm.

^{13}C-NMR (101 MHz, CD_2Cl_2): δ = 154 (s, C_e), 150 (s, C_a), 150 (s, C_i), 146 (s, C_c), 141 (s, C_f), 130 (s, C_k), 121 (d, C_n), 115 (d, C_b), 111 (d, C_d), 110 (d, C_h), 109 (d, C_l), 15.2 (q, C_g), 13.5 (q, C_j) ppm.

IR (ATR): $\tilde{\nu}$ = 3470 (w), 3102 (w), 2969 (w), 2919 (w), 1554 (s), 1480 (w), 1464 (w), 1440 (m), 1402 (s), 1373 (m), 1303 (m), 1271 (w), 1243 (w), 1180 (w), 1136 (w), 1096 (m), 1054 (m), 1039 (m), 970 (m), 946 (w), 883 (w), 854 (m), 814 (m), 780 (s), 728 (s), 685 (w), 659 (w), 640 (w), 622 (w), 606 (m), 550 (s), 427 (w), 398 (m), 369 (w), 308 (w), 252 (w), 199 (m), 146 (w), 118 (m), 92 (s), 62 (s) cm^{-1}.

FT-RAMAN (KBr): $\tilde{\nu}$ = 1572 (w), 1468 (w), 1441 (w), 987 (w) cm^{-1}.

HR-ESI-MS (-): m/z = 271.07541 (calc. 271.0745).

Anal. Calcd for $C_{14}H_{13}N_4Cl$: C, 61.65 %; H, 4.80 %; N, 20.54 %. Found: C, 61.59 %; H, 4.65 %; N, 20.21 %.

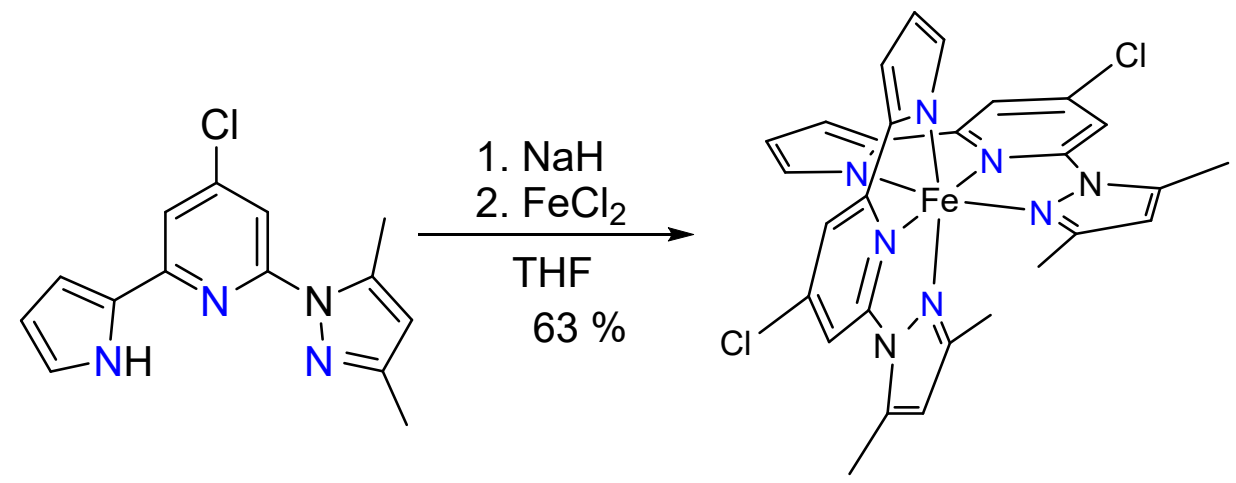

2.5. [Fe(pzpypyr-Cl)$_2$]

To a solution of the ligand pzpypyr-Cl (815 mg, 2.99 mmol, 1 eq) in 40 ml abs. THF at 0 °C NaH (60 % dispersion in mineral oil, 143.4 mg, 3.59 mmol, 1.2 eq) was added in small portion in N_2 counterflow. The solution turned yellow and was stirred for 2 h at 0 °C. Then 199.8 mg $FeCl_2$ (1.49 mmol, 0.5 eq) in 5 ml abs. THF was added and rinsed with another 10 ml abs. THF. The solution turned dark red. The mixture was allowed to warm to room temperature and stirred for 3 h. The raw product was filtered over Celite before the solvent was removed *in vacuo*. The resulting solid was washed with 70 mL cold abs. diethyl ether to obtain the product as a red powder with a yield of 566 mg (945 μmol, 63 %). Crystals suitable for single crystal analysis were obtained by vapor diffusion of *n*-hexane in THF.

^{1}H-NMR (400 MHz, CD_2Cl_2): δ = 13.5 (br. s, 2 H, H_{py}), 12.1 (br. s, 2 H, H_{py}), 11.6 (br. s, 2 H, H_{pyr}, 10.6 (br. s, 2 H, H_{pyr}), 10.4 (br. s, 2 H, H_{pyr}), 7.15 (br. s, 2 H, H_{pz}), 2.71 (br. s, 6 H, $H_{\mathrm{pz\text{-}me}}$), 1.68 (br. s, 6 H, $H_{\mathrm{pz\text{-}me}}$) ppm.

IR (ATR): $\tilde{\nu}$ = 3101 (w), 2922 (w), 1592 (s), 1569 (w), 1535 (w), 1513 (s), 1456 (m), 1427 (s), 1373 (m), 1293 (m), 1245 (br. m), 1198 (w), 1151 (w), 1109 (m), 1084 (m), 1033 (br. s), 982 (m), 953 (m), 830 (m), 781 (s), 739 (m), 716 (s), 686 (w), 685 (w), 603 (b. w), 562 (w), 542 (m), 505 (w), 393 (w), 261 (b. w), 126 (w), 87.8 (s), 67 (w) cm^{-1}.

FT-RAMAN (KBr): $\tilde{\nu}$ = 2920 (w), 1594 (m), 1568 (w), 1544 (w), 1514 (m),1475 (m), 1414 (w), 1377 (m), 1315 (m), 1296 (m), 1059 (w), 1030 (m), 999 (m), 955 (w) cm^{-1}.

HR-ESI-MS (+): m/z = 599.09137 (calc. 599.09232).

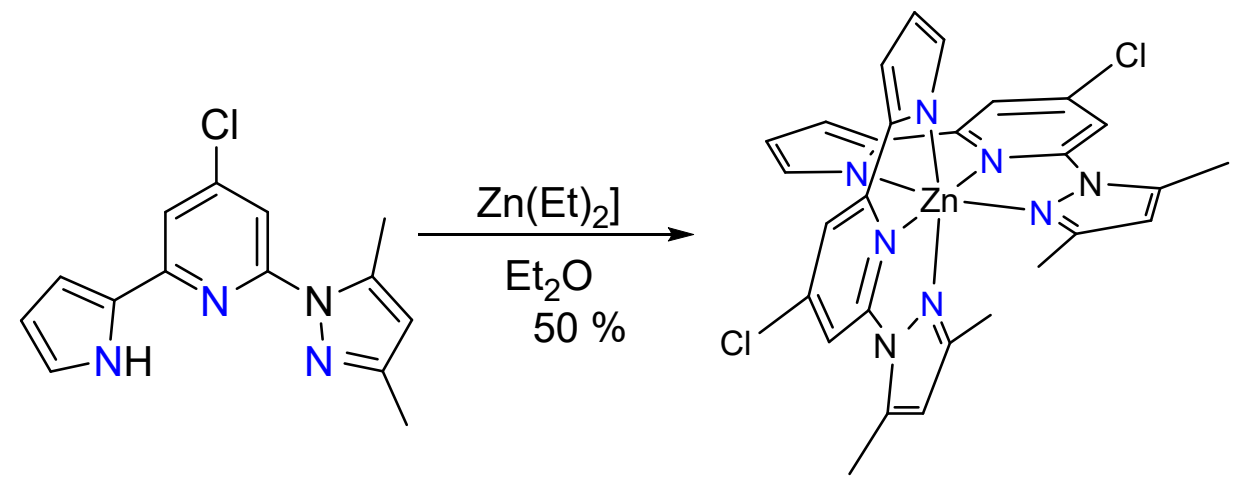

2.6. [Zn(pzpypyr-Cl)$_2$]

The zinc complex was prepared after a protocol from the literature.[6] The ligand pzpypyr-Cl (101 mg, 369 μmol, 1 eq) was dissolved in 5 ml abs. diethyl ether and cooled to -96 °C. Then Et_2Zn (0.18 mL, 1 M in *n*-hexane, 180 μmol, 0.5 eq) was slowly added. The yellow mixture was stirred for 40 min at −96°C and 16 min at room temperature, during which a yellow precipitate was formed. The mixture was filtered, washed with 20 ml abs. Et_2O and dried *in vacuo* at 80°C for 4 h. The product was obtained as a yellow solid in a yield of 68.8 mg (113 μmol, 31 %). Crystals suitable for single crystal analysis were obtained by vapor diffusion of *n*-hexane in THF.

^{1}H-NMR (400 MHz, CD_2Cl_2): δ = 7.30 (d, ^4J = 1.5 Hz, 1 H, H_d), 6.83 (dd, ^3J = 3.2 Hz, ^4J = 1.1 Hz,1 H, H_n), 6.80 (d, ^4J = 1.5 Hz, 1 H, H_b), 6.66 (dd, ^3J = 1.6 Hz,^4J = 1.1 Hz, 1 H, H_l), 6.20 (dd, ^3J = 3.4, 1.6 Hz, 1 H, H_m), 5.81 (dd, ^4J = 0.9, 0.5 Hz, 1 H, H_h), 2.37 (s, 3 H, H_g), 1.6 (d, ^4J = 0.8 Hz, 3 H, H_j) ppm.

IR (ATR): $\tilde{\nu}$ = 3154 (w), 3101 (w), 3063 (w), 2922 (w), 1597 (s), 1548 (s), 1515 (s), 1480 (w), 1450 (w), 1416 (s), 1374 (m), 1363 (m), 1320 (m), 1281 (s), 1258 (w), 1197 (m), 1171 (w), 1150 (m), 1110 (m), 1093 (w), 1036 (s, arom. C-Cl-Val.), 991 (m), 952 (m), 897 (w), 844 (m), 821 (s), 780 (s), 745 (s), 727 (s), 694 (w), 637 (w), 610 (m), 566 (m), 515 (w), 446 (w), 394 (m), 344 (w), 273 (m), 213 (m), 174 (s), 123 (w), 100 (w), 81 (s), 64 (s) cm^{-1}.

FT-RAMAN (KBr): $\tilde{\nu}$ = 3107 (w), 2924 (w), 1571 (w), 1595 (m), 1570 (m), 1549 (m), 1520 (s),1416 (w), 1390 (s), 1377 (s), 1294 (m), 1200 (w), 1151 (w), 1113 (w), 1095 (w), 1069 (w), 1306 (w), 1001 (s), 955 (w), 783 (w), 723 (w), 347 (w), 274 (w), 251 (w), 187 (w), 79 (m) cm^{-1}.

HR-ESI-MS (+): $m/z = 607.09427$ (calc. 607.08652).

3. Structural and Spectroscopic Characterization

3.1. Mössbauer Spectra of [Fe(pzpypyr-Cl)$_2$]

Mössbauer spectra were recorded on a WISSEL Mössbauer setup equipped with an Oxford cryostat.

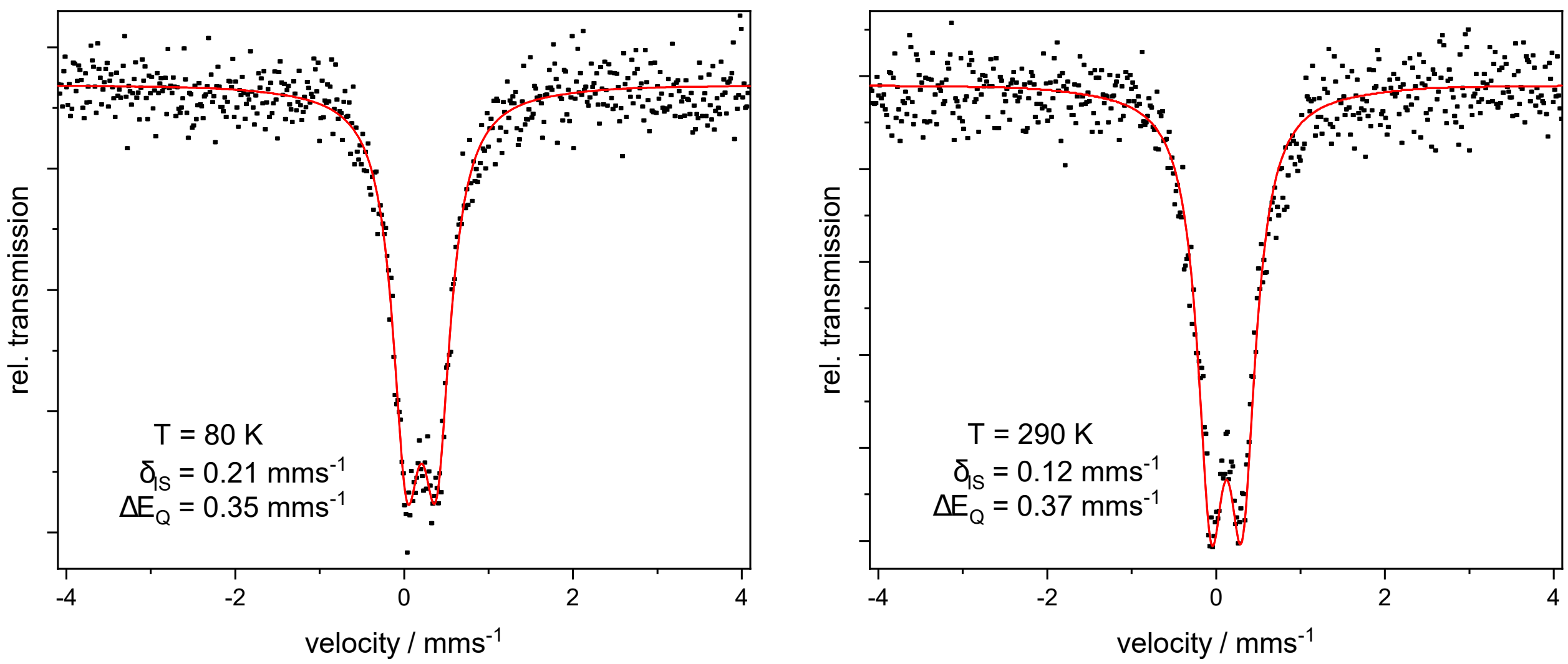

Figure S1: **Mössbauer spectra of [Fe(pzpypyr-Cl)$_2$] at 80 and 290 K.** The respective isomer shift δ and quadrupole splitting ΔEQ is given.

3.2. Single-Crystal Structures

Data collection was performed with a XtaLAB Synergy, Dualflex, HyPix diffractometer. The structure was solved with SHELXT and refined with SHELXL using least-squares minimisation. All non-hydrogen atoms were refined anisotropically. The C-H H atoms were positioned with idealized geometry (methyl H atoms allowed to rotate but not to tip) and were refined isotropic with Uiso(H) = 1.2 Ueq(C) (1.5 for methyl H atoms) using a riding model. The

asymmetric unit of [Fe(pzpypyr-Cl)$_2$] contains in maximum one dichloromethane molecule that is disordered. Because no reasonable structure model was found, its contribution to the electron density was removed. The carbon-atoms C10 C11 C12 C13 were refined using restraints (RIGU). CCDC-2571355 and CCDC-2571356 contain the supplementary crystallographic data for this paper. These data can be obtained free charge from the Cambridge Crystallographic Data Centre via https://www.ccdc.cam.ac.uk/data_request/cif.

3.3. Crystal Structures of [M(pzpypyr-Cl)$_2$], M = Fe, Zn

The ligand pzpypyr-Cl was designed with the help of calculated structures, to form complexes, that allow for linear chains to be created. During screening, we looked at the angle spanned between the Fe(II) center and the C-atoms carrying the chlorine substituents (C_c in the NMR-assignments) ($\varphi = \angle$(Pyridine-C_4–Fe–Pyridine-C_4). This angle φ would ideally change very little upon the spin transition. In place of a high-spin structure of [Fe(pzpypyr-Cl)$_2$], we used the crystal structure of [Zn(pzpypyr-Cl)$_2$]. Indeed, $\Delta\varphi$ was found to be small (2.6°; cf Table S1. However, upon going from low-spin [Fe(pzpypyr-Cl)$_2$] to the putative high-spin (*i.e.* Zn(II)) structure, a structural change occurs at the pyrazole ring, which bends out of the ligand plane. This effect can be described by the angle θ between the coordinating N-atoms of the pyrazole units, the metal center and the *C*H atoms (C_h in the NMR-assignments) of the pyrazole units ($\theta = \angle$(Pyrazole-N_{coord}–Fe–Pyrazole-*C*H)). The latter distortion, however, does not influence the linearity of the chain. Relevant structural parameters (bond lengths, bond angles of [Fe(pzpypyr-Cl)$_2$] and [Zn(pzpypyr-Cl)$_2$]) are collected in Table S1.

Table S1: bond lengths and bond angles in [Fe(pzpypyr-Cl)$_2$] and [Zn(pzpypyr-Cl)$_2$].

Bond lengths or angles in [M(pzpypyr-Cl)$_2$]	M = Fe/Å or °	M = Zn/Å or °
N_{Py1}-M	1.895(5)(N_3)	2.169(4)(N_{102})
N_{Py2}-M	1.892(5)(N_{24})	2.161(4)(N_{202})
N_{Pyr1}-M	1.961(5)(N_1)	2.046(4)(N_{101})
N_{Pyr2}-M	1.964(4)(N_{23})	2.088(4)(N_{201})
N_{Pz1}-M	1.988(4)(N_{21})	2.286(4)(N_{104})
N_{Pz2}-M	1.961(5)(N_4)	2.266(4)(N_{204})
φ	169.8°	167.2°
$\Delta\varphi$	2.6°	
θ	2.5° and 1.8°	11.3° and 7.2°

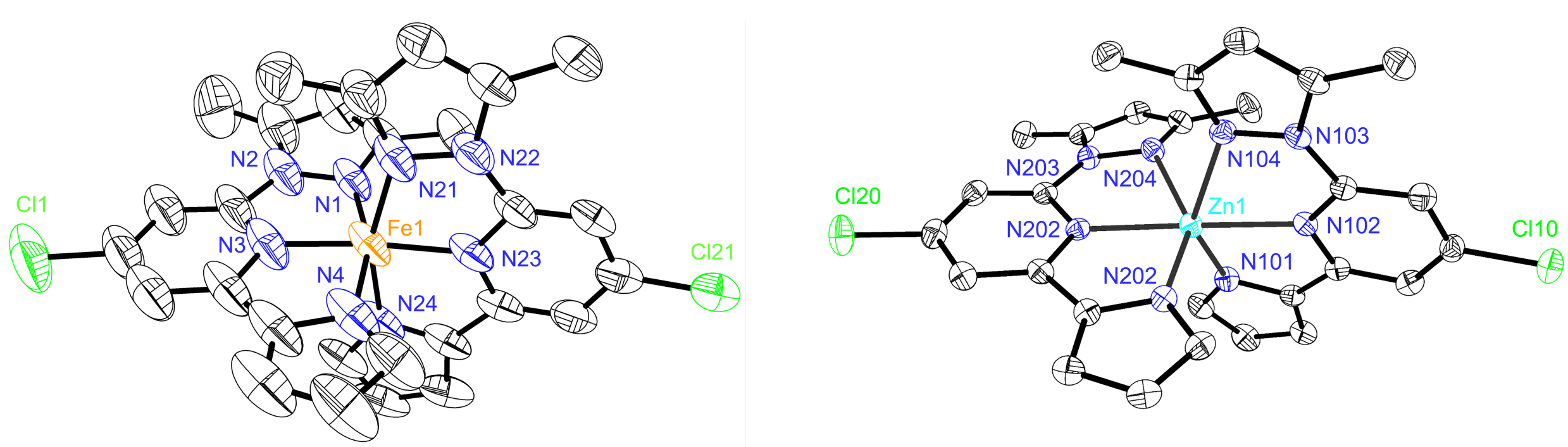

Figure S2: **ORTEP type figures of [Fe(pzpypyr-Cl)$_2$]and [Zn(pzpypyr-Cl)$_2$]**. In theory an isomeric structure, in which the right handed ligand would be rotated by 180°, would be possible, but this isomer was not found in either of the structures. Anisotropic displacement ellipsoids are shown at the 50 % probability level.

Crystal data of [Fe(pzpypyr-Cl)$_2$] and [Zn(pzpypyr-Cl)$_2$]

Table S2: Crystal data and structure refinement for [Fe(pzpypyr-Cl)$_2$]

Empirical formula	$C_{28}H_{24}Cl_2N_8Fe$
Formula weight	599.30
Temperature/K	100.00(13)
Crystal system	trigonal
Space group	$P3_221$
a/Å	14.4280(3)
b/Å	14.4280(3)
c/Å	26.2580(3)
α/°	90
β/°	90
γ/°	120
Volume/Å^3	4733.74(19)
Z	6
ρ_{calc}g/cm^3	1.261
μ/mm^{-1}	5.627
F(000)	1848.0
Crystal size/mm^3	0.4 × 0.02 × 0.02
Radiation	Cu Kα (λ = 1.54184)
2Θ range for data collection/°	7.074 to 160.638
Index ranges	$-18 \leq h \leq 17$, $-17 \leq k \leq 17$, $-22 \leq l \leq 31$
Reflections collected	26027
Independent reflections	6552 [R_{int} = 0.0280, R_{sigma} = 0.0236]
Data/restraints/parameters	6552/15/356
Reflections with I > 2 σ (I)	5475
Goodness-of-fit on F^2	1.064
Final R indexes [I≥2σ (I)]	R_1 = 0.0542, wR_2 = 0.1579
Final R indexes [all data]	R_1 = 0.0627, wR_2 = 0.1661
Largest diff. peak/hole / e Å^{-3}	0.52/-0.39
Flack parameter	-0.013(4)

Table S3: Crystal data and structure refinement for $[Zn(pzpypyr\text{-}Cl)_2]$

Empirical formula	$C_{28}H_{24}Cl_2N_8Zn$
Formula weight	608.82
Temperature/K	100.00(13)
Crystal system	triclinic
Space group	P-1
a/Å	8.4700(7)
b/Å	10.1450(5)
c/Å	16.3545(7)
α/°	72.582(4)
β/°	87.350(5)
γ/°	84.628(5)
Volume/$Å^3$	1334.74(14)
Z	2
ρ_{calc}g/cm^3	1.515
μ/mm^{-1}	3.399
F(000)	624.0
Crystal size/mm^3	$0.1 \times 0.05 \times 0.01$
Radiation	Cu Kα ($\lambda = 1.54184$)
2Θ range for data collection/°	5.664 to 159.926
Index ranges	$-10 \leq h \leq 10$, $-9 \leq k \leq 12$, $-20 \leq l \leq 20$
Reflections collected	15630
Independent reflections	5562 [$R_{int} = 0.0426$, $R_{sigma} = 0.0442$]
Data/restraints/parameters	5562/0/357
Reflections with I < 2 σ (I)	4927
Goodness-of-fit on F^2	1.056
Final R indexes [I≥2σ (I)]	$R_1 = 0.0610$, $wR_2 = 0.1601$
Final R indexes [all data]	$R_1 = 0.0688$, $wR_2 = 0.1649$
Largest diff. peak/hole / e Å^{-3}	0.95/-0.86

4. Additional STM and STS Results

4.1. Topograph of Chains

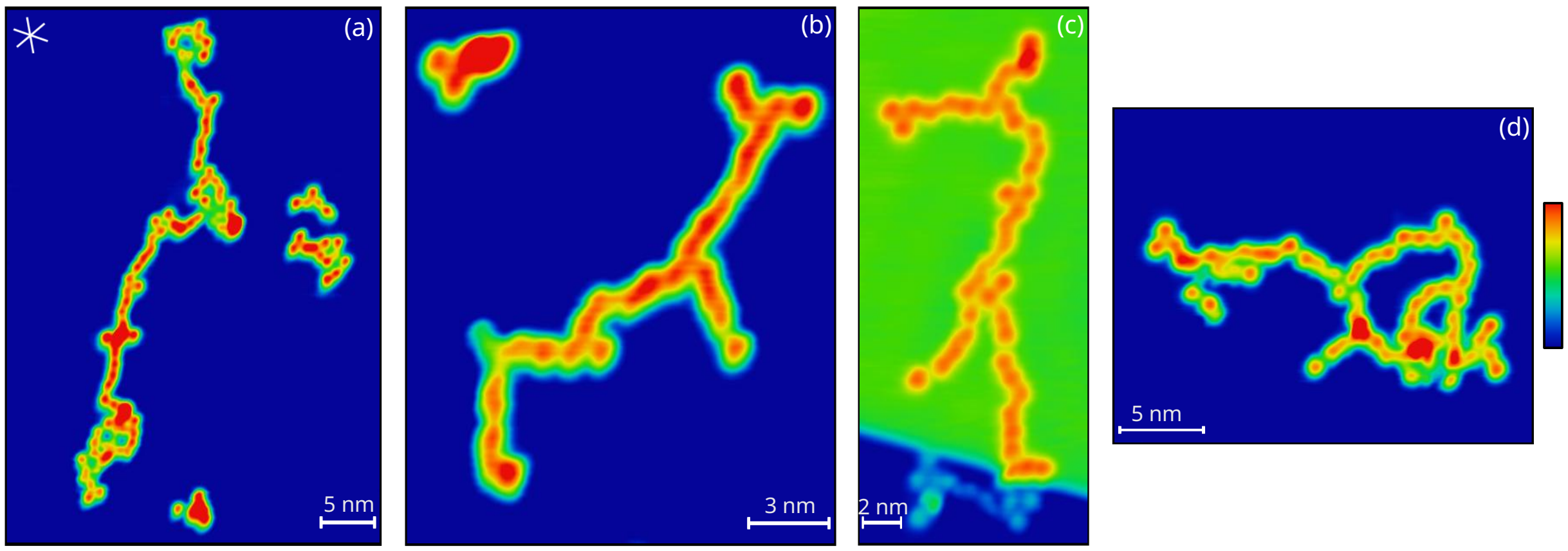

Figure S3: **Gallery of chains prepared from [Fe(pzpypyr-Cl)$_2$].** Topographs of a few chains obtained by annealing. Imaging parameters: $I = 5\,\mathrm{pA}$ and (a) -0.1, (b) -0.2, (c) -0.2, and (d) $-0.5\,\mathrm{V}$. Image areas: 35×50 , 16×20 , 12×28 , and $25 \times 20\,\mathrm{nm}^2$, respectively. The white lines in panel (a) indicate the close-packed directions of the Ag(111) substrate. The false colors in (a – d) cover height ranges of 120, 130, 420, and 180 pm, respectively.

4.2. Three State Switching in $I(V)$

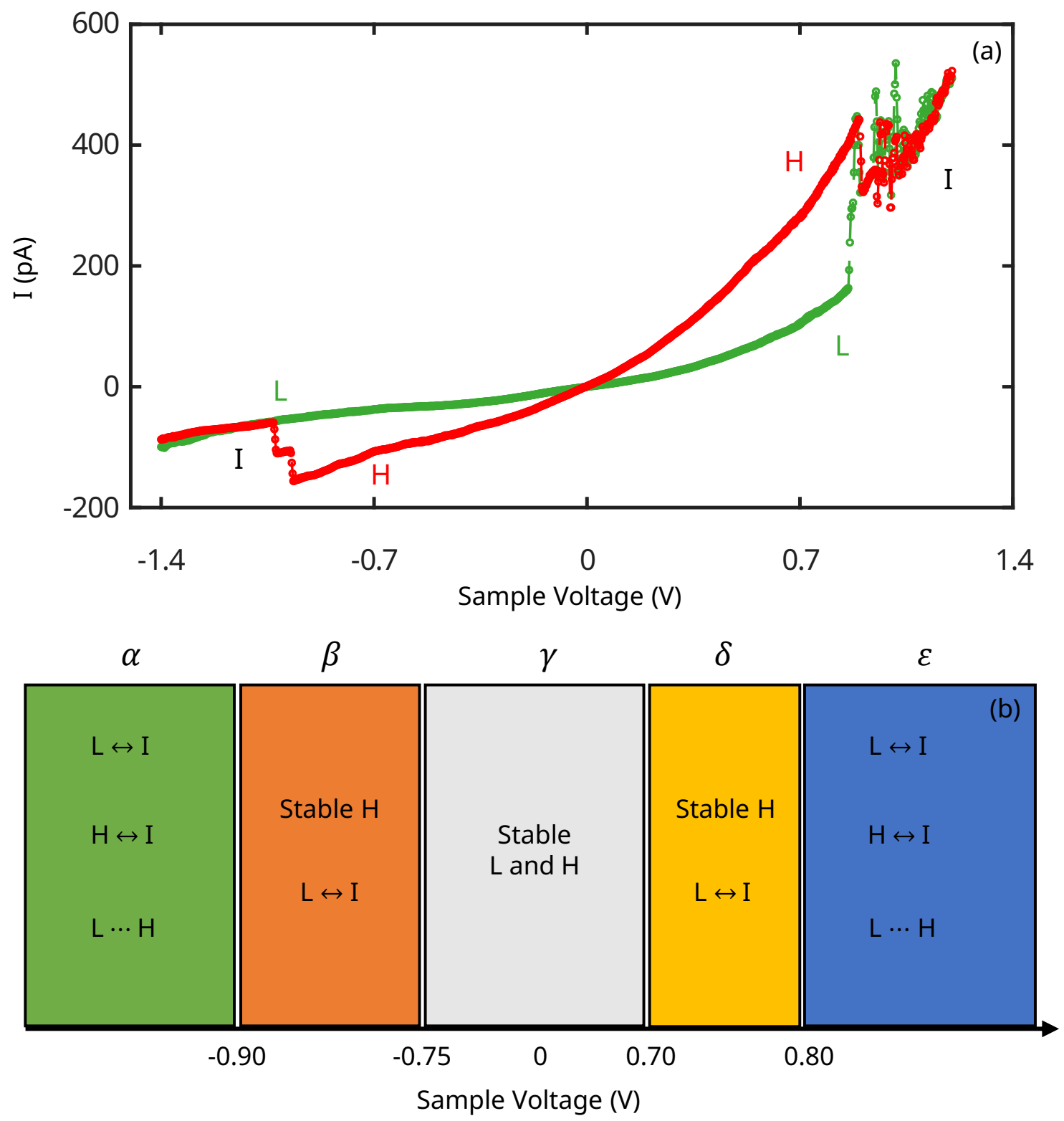

Figure S4: **Three state switching in current-voltage characteristics.** (a) Current-voltage characteristics of a switchable complex adsorbed at a step edge of the Ag(111) substrate. Green and red curves correspond to the forward and backward voltage sweeps, respectively, between -1.4 and 1.3 V. In the voltage range from -0.95 to 0.85 V, two stable states (L and H) are observed. An intermediate state (I) emerges outside this range. Prior to the voltage sweep, the current feedback loop was opened at -1.4 V and 100 pA. (b) Schematic illustration summarizing the stability and switching behavior. The L and H states are stable between -0.75 and 0.70 V. In the ranges from -0.75 to -0.90 V and $+0.70$ to $+0.80$ V, transitions between the L and I states occur (double headed arrow). At higher bias magnitudes, transitions between L $\leftrightarrow$ I and H $\leftrightarrow$ I occur (double sided arrow), while direct transitions between L and H are less frequent (dotted lines). Note that, x-axis [Sample Voltage (V)] is not to scale.

4.3. $I(V)$ of the Far From Step-Edge Molecule

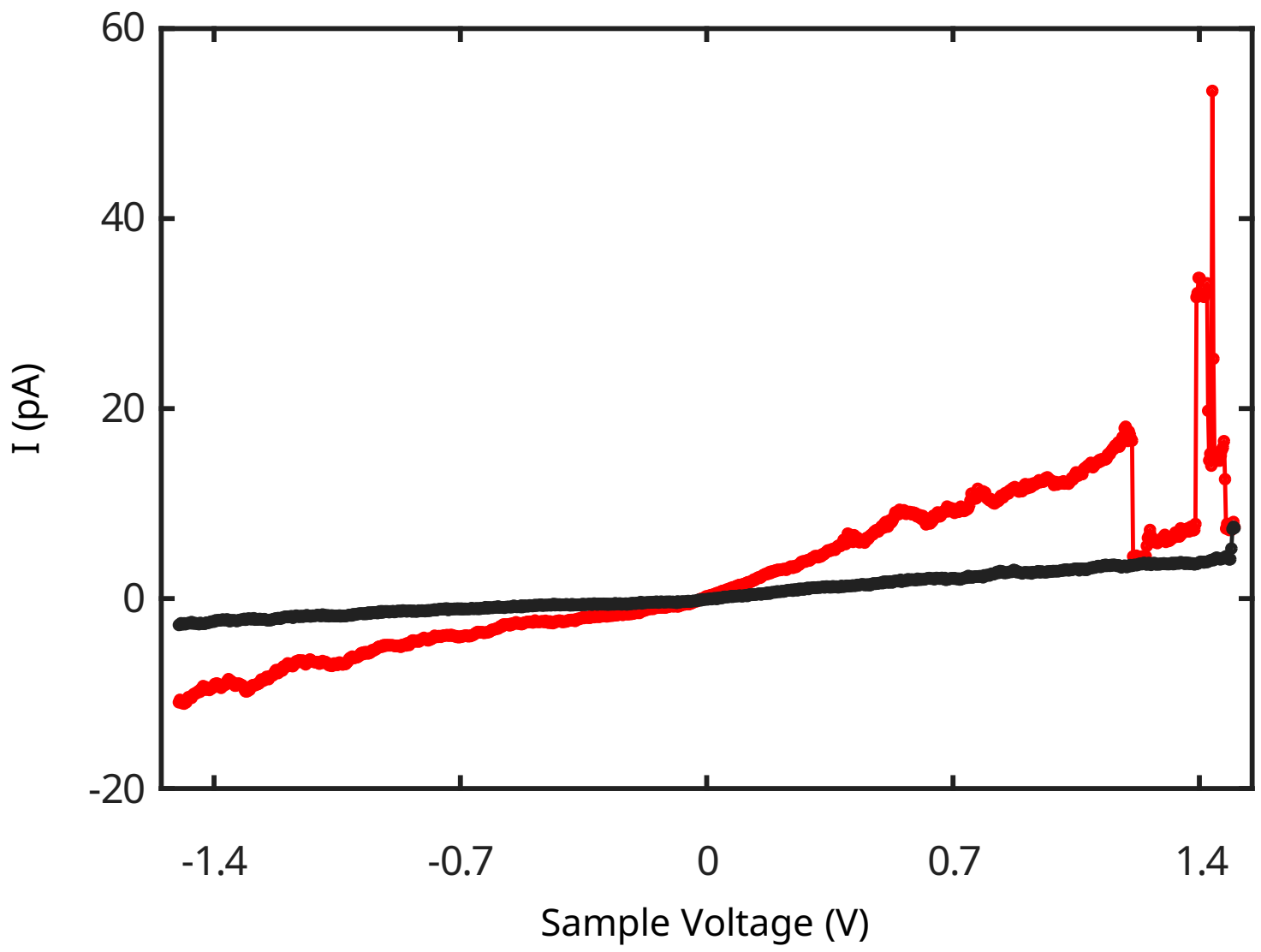

Figure S5: **Current-voltage characteristics.** Current-voltage characteristics of molecule adsorbed on a terrace of the Ag(111) substrate. Red and black dots indicate datapoints from the forward and backward voltage sweeps. Prior to the measurement, the current feedback loop was opened at $-1.5\,\mathrm{V}$ and $10\,\mathrm{pA}$.

4.4. Current-Induced Rotation

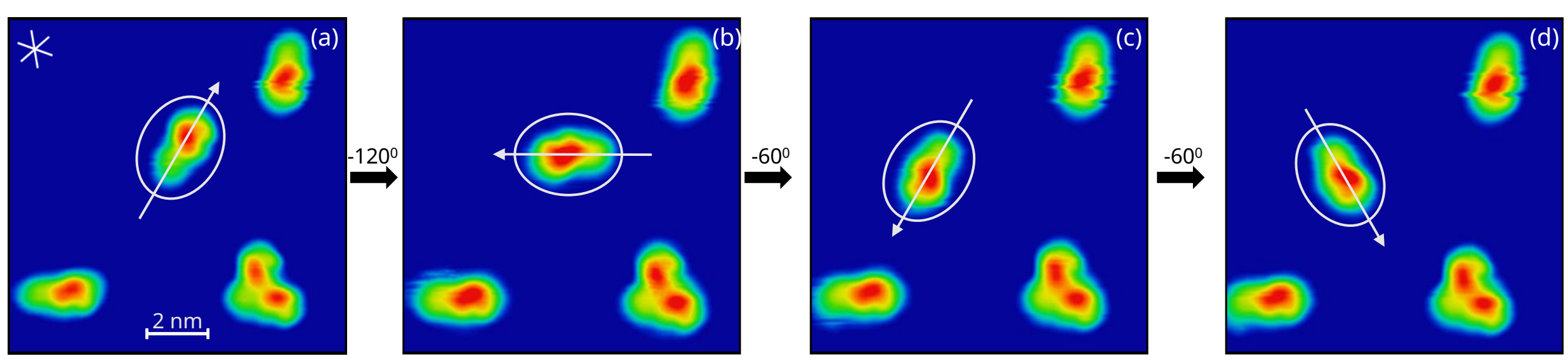

Figure S6: **Rotation induced by voltage pulses.** Sequence of topographs ($-0.5\,\mathrm{V}$, $5\,\mathrm{pA}$, $11 \times 11\,\mathrm{nm}^2$). Between frames, voltage pulse were applied to the complex marked by an ellipse. While the shape of the corresponding image feature remains largely unchanged, rotations by multiples of 60° occur. White lines mark the close-packed directions of the Ag(111) substrate. The false colors in (a – d) cover height range of 220 pm.

4.5. Line Profiles

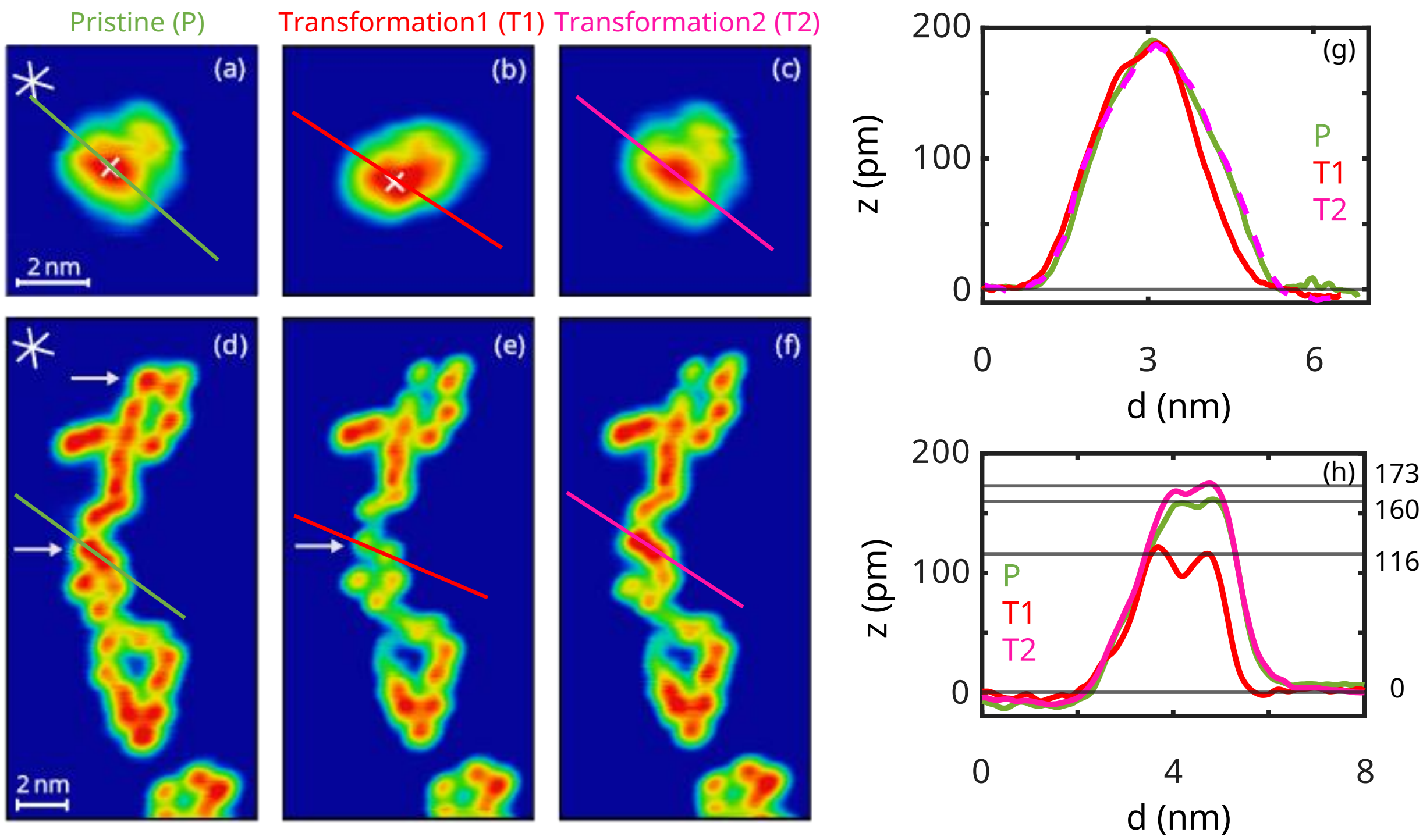

Figure S7: **Line profiles.** (a – c) and (d – f) reproduce the topographs shown in Figure 5 of the main text. The line profiles shown in (g) and (h) were measured along the paths marked in matching colors.

4.6. Low Bias Excitations

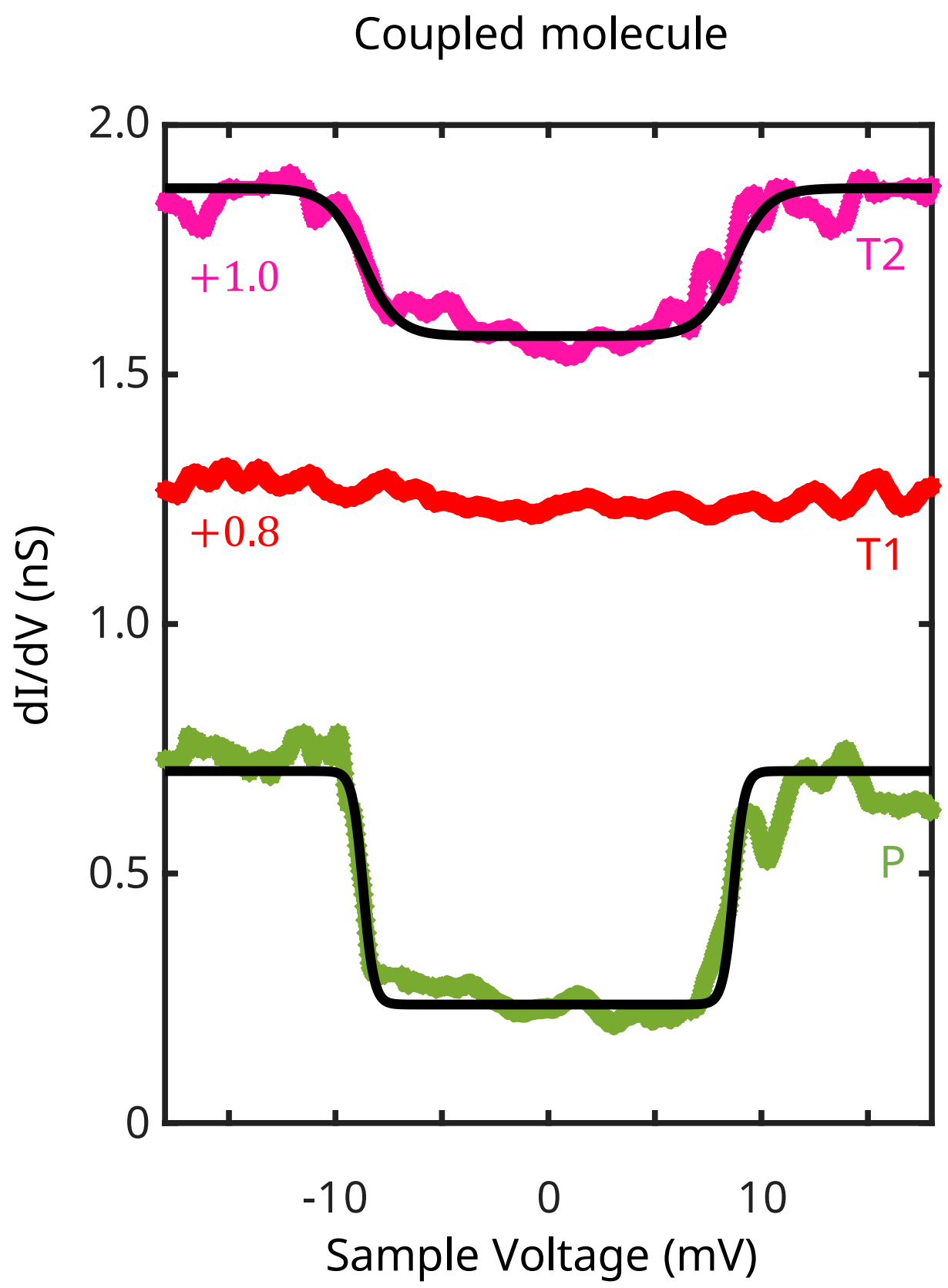

Figure S8: **Fit of spin excitations with ferromagnetic interaction.** The spectra shown in Figure 6b of manuscrpt can also be fitted using a ferromagnetic exchange coupling $J = 1.45\,\text{meV}$.

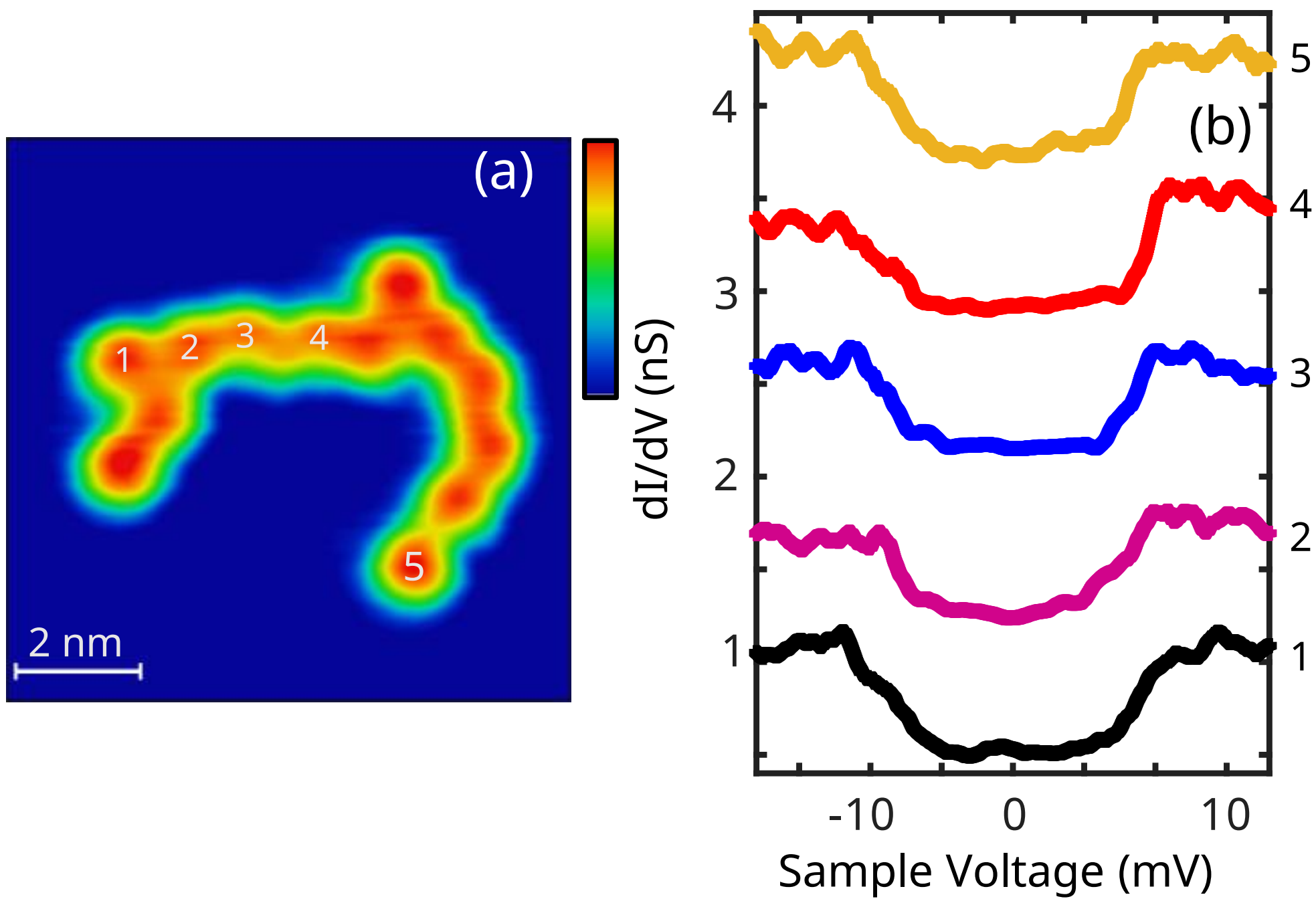

Figure S9: **Spin-flip excitation spectra in a molecular spin chain.** (a) Constant-current STM topograph of a molecular chain on Ag(111) ($9 \times 9\,\mathrm{nm}^2$, $V = -0.5\,\mathrm{V}$, $I = 5\,\mathrm{pA}$). The false colors cover a height range of 150 pm. Five molecules are labelled with numbers. (b) Differential conductance spectra acquired above the centers of the labelled molecules, measured at 4.2 K in the absence of an external magnetic field. For clarity, the spectra are vertically offset by multiples of 0.80 nS. Prior to recording $\mathrm{d}I/\mathrm{d}V$, the current feedback loop was opened at $V = -20\,\mathrm{mV}$ and $I = 200\,\mathrm{pA}$.

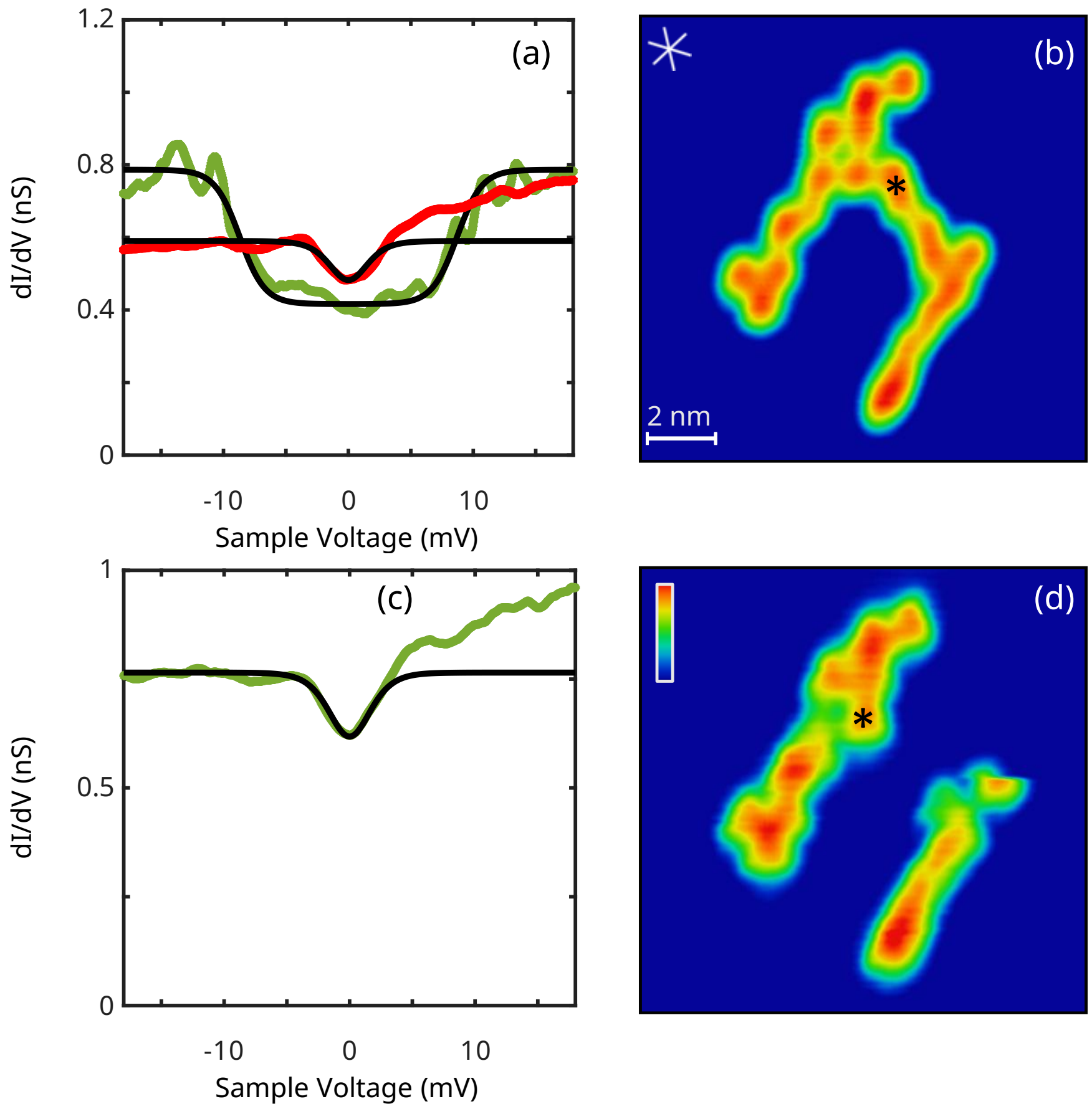

Figure S10: **Differential conductance spectra using a functionalized tip.** (a) $\mathrm{d}I/\mathrm{d}V$ spectra measured at the position marked with an asterisk in (b). The green and red lines correspond to upward and downward voltage sweeps. During the backward sweep, molecules were removed from the surface, as confirmed by subsequent imaging (d). This tip functionalization leads to modified conductance spectra. (c) Spectrum acquired with a functionalized tip, measured on top of a molecule on the substrate as indicated by $'*'$ in (d). Black lines in (a) show fits using the Hamiltonian in Eq. (1) of the main text, whereas black lines corresponding to red plot in a and in c was modelled using Hamiltonian, $H = DS_z^2 + E(S_x^2 - S_y^2)$. D and E are single ion anisotropy parameters. Parameters used: $J = -2.9\,\mathrm{meV}$ for the green curve in a, $D = 0.20\,\mathrm{meV}$ and $E = 0.05\,\mathrm{meV}$ from gas phase calculations for the red and green curves in a and c. The false colors in panels (b) and (d) cover height ranges of 140, and 130 pm, respectively.

4.7. Control experiments using [Zn(pzpypyr-Cl)$_2$]

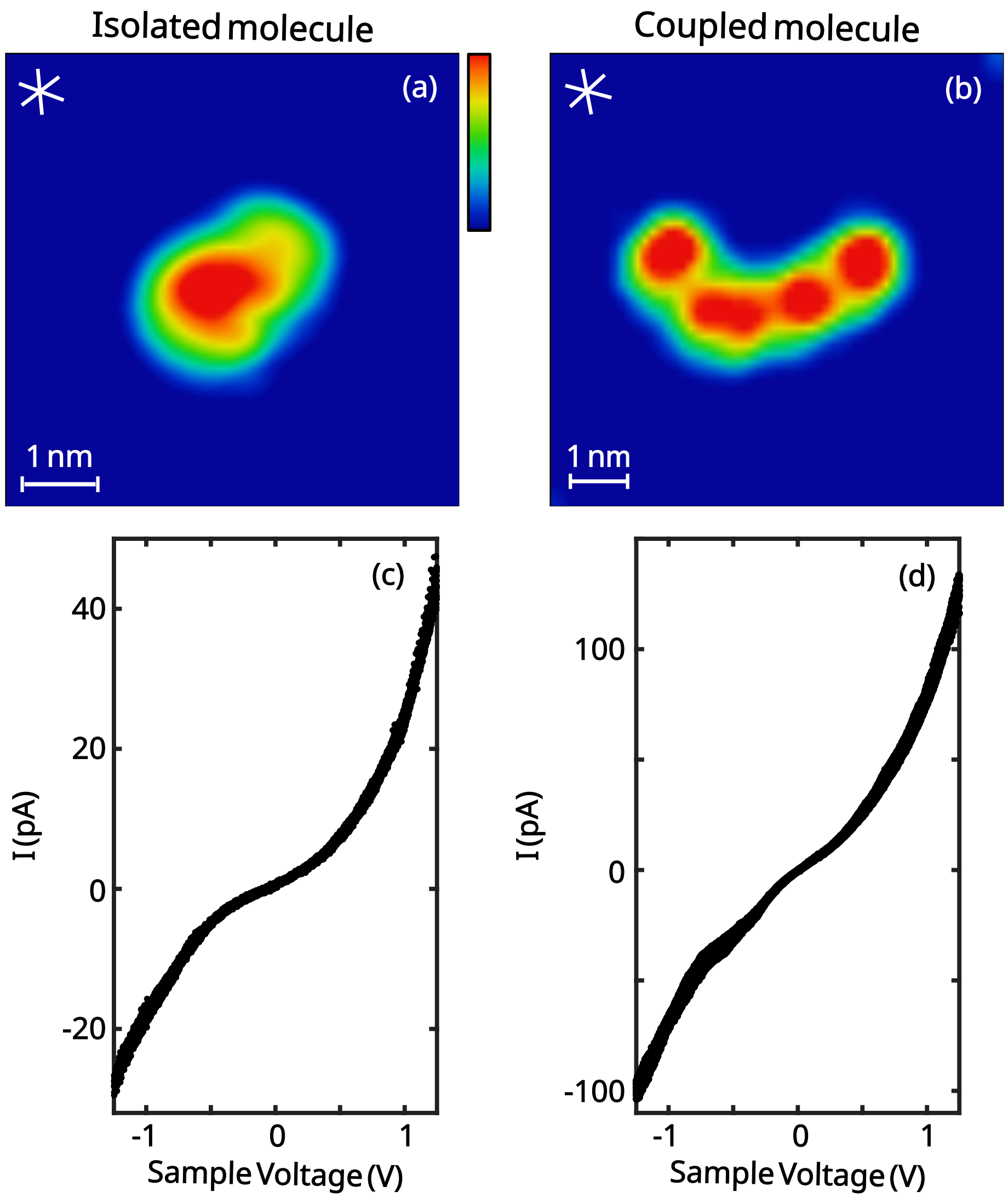

Figure S11: **Control experiment with [Zn(pzpypyr-Cl)$_2$] on Ag(111).** Topographs of (a) an isolated complex and (b) a molecular chain. Imaged areas: (6 nm)^2 and (8 nm)^2. White lines indicate the densely packed directions of the Ag(111) substrate. The false colors cover height ranges of (a) 180 and (b) 100 pm. (c, d) $I(V)$ characteristics of an isolated complex and a complex in a chain, respectively. In each case, data from ten consecutive voltage sweeps from −1.5 to 1.5 V and back are displayed. No evidence of bistability is observed. Prior to the voltage sweeps, the current feedback loop was opened at −1.5 V and 50 pA (150 pA) for the isolated molecule (molecule in the chain).

5. Modelling

5.1. Model of Chain Segments

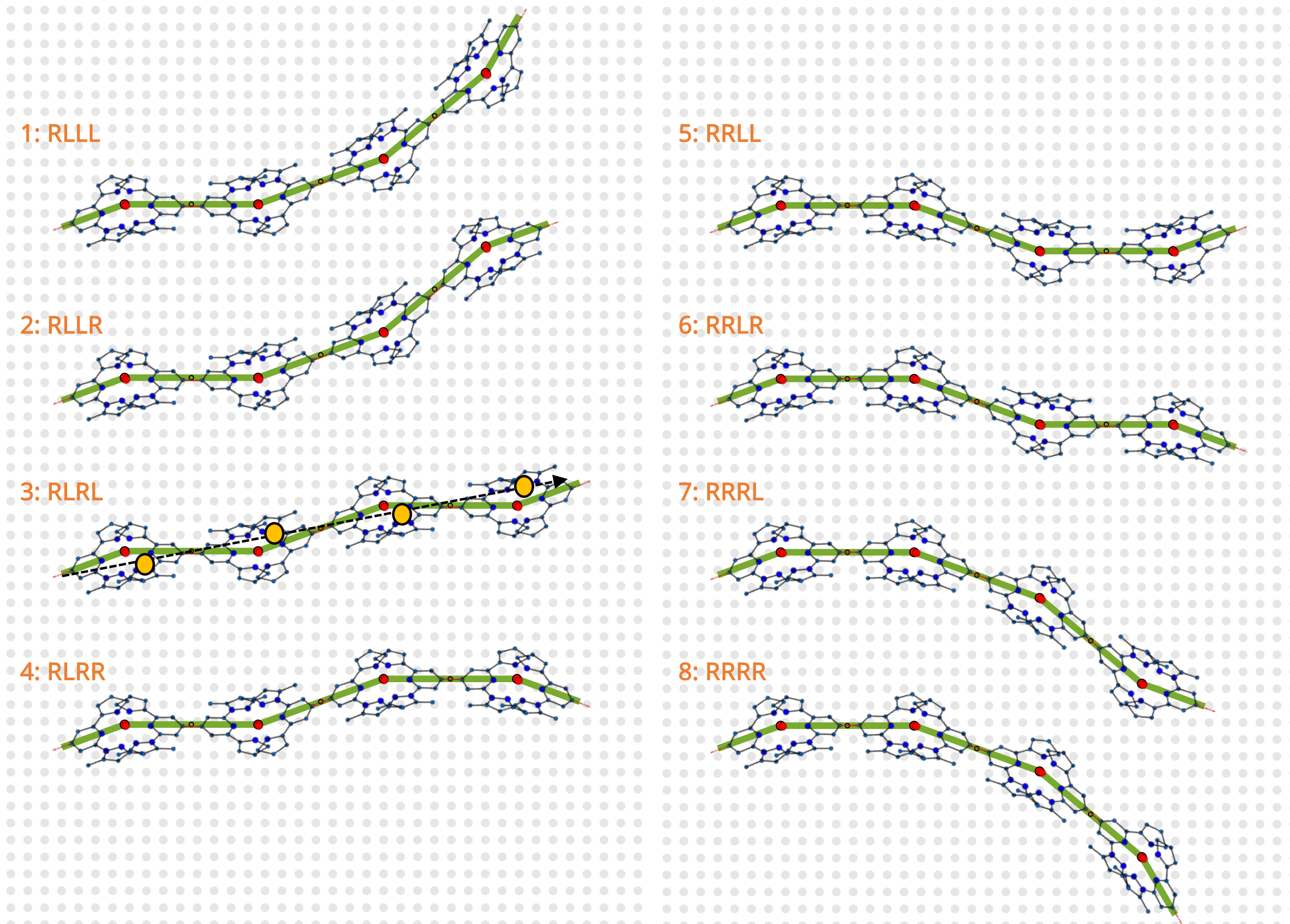

Figure S12: **Proposed models of tetramer chains.** The leftmost molecule is assumed to be adsorbed in an R state. The other molecules are either R or L leading to 8 conceivable variations of the chain structure. The green line serves as a visual guide for the chain trajectory, where one ligand of each molecule is rotated by 20° around the central Fe atom (red) with respect to the other ligand, either clockwise (R) or counterclockwise (L). Gray circles represent the Ag substrate, and the red dashed lines denote the covalent coupling between adjacent molecules. For configuration 3 (RLRL), a straight chain is expected (black dashed line), with yellow dots highlighting the positions where maximum imaging intensity is predicted by our DFT calculations for isolated molecules.

5.2. DFT Modelling

We performed DFT calculations (see details in the Methods section) to model the [Fe(pzpypyr-Cl)$_2$] complex in various configurations, starting with the free molecule in the gas phase. As expected, the low-spin (LS) state is 45 meV lower in energy than the high-spin (HS) state, and the two flat ligands, connected via the iron atom, are nearly perpendicular to each other.

The next, more challenging step is determining the adsorption geometry. We focused on configurations where the Cl-Fe-Cl direction is parallel to the surface plane and approximately aligned with the x axis – the dense direction of Ag(111) (see Computational Details in the Methods section). We then considered three plausible adsorption geometries, depending on the orientation of the pyrazole rings (connected to two methyl groups) relative to the silver substrate. In the $Pz_{up,up}$ and $Pz_{dn,dn}$ configurations, both pyrazole rings point toward the vacuum or substrate, respectively, while $Pz_{up,dn}$ represents the intermediate configuration (see Figure S13)

Geometry optimization of the three configurations for both low-spin (LS) and high-spin (HS) states shows that $Pz_{up,up}$ is the most favorable. The energy difference between $Pz_{up,up}$

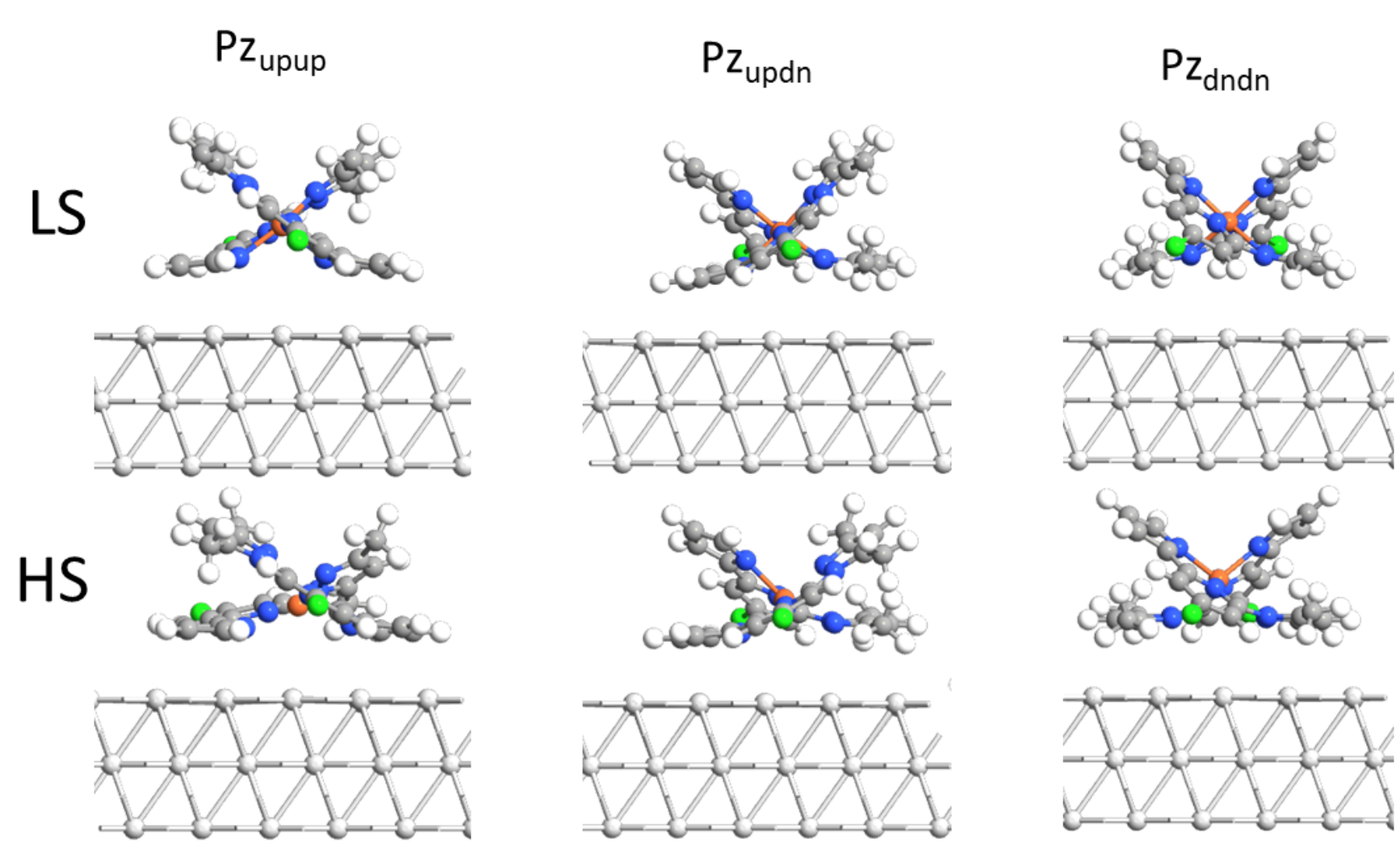

Figure S13: The three conformers considered, $\mathrm{Pz_{up,up}}$, $\mathrm{Pz_{up,dn}}$ and $\mathrm{Pz_{dn,dn}}$, of [Fe(pzpypyr-Cl)$_2$] in the LS and HS states on Ag(111). The most stable structure is $\mathrm{Pz_{up,up}}$ in the high-spin state. The x-axis is pointing out of the plane.

and the other two configurations is enhanced in the HS state (with $Pz_{dn,dn}$ always being the least favorable). The HS state is strongly favored, with an energy 350 meV lower than the LS state. The relaxation also reveals significant molecular flattening, which stabilizes the HS state due to its greater flexibility. Additionally, while the Cl-Fe-Cl direction remains roughly aligned with the x-axis, a clear distortion of the complex emerges: the iron atom is no longer aligned with the chlorine atoms, creating a bending of the molecule either to the left (L) or right (R). This bending is particularly pronounced when [Fe(pzpypyr-Cl)$_2$] is in the HS state. This distorsion is the consequence of the influence of van der Waals interaction that strongly favors pyridine rings as flat as possible on the silver surface. This can only be achieved by breaking of the symmetry and bending the Cl-Fe-Cl axis.

Starting from this first relaxed structure, we applied a 30° rotation about the z axis and performed a new geometry optimization. Interestingly, the molecule in the LS state returns to its original configuration with the Cl-Fe-Cl direction approximately along the $x$axis, while in the HS state, a new local minimum was found with an energy 80 meV lower than the x-aligned configuration. A top-view of the lowest energy structures for LS and HS states are shown in Figure 3 of the manuscript.

Based on the proposed model of chain segments we have built the shortest straight periodic chain RL with 2 molecules per unit-cell (Figure S14). The geometry of the structure was then optimized keeping the periodic boundary conditions fixed with a lattice vector of 2.1 nm and ignoring the silver substrate for numerical reasons.

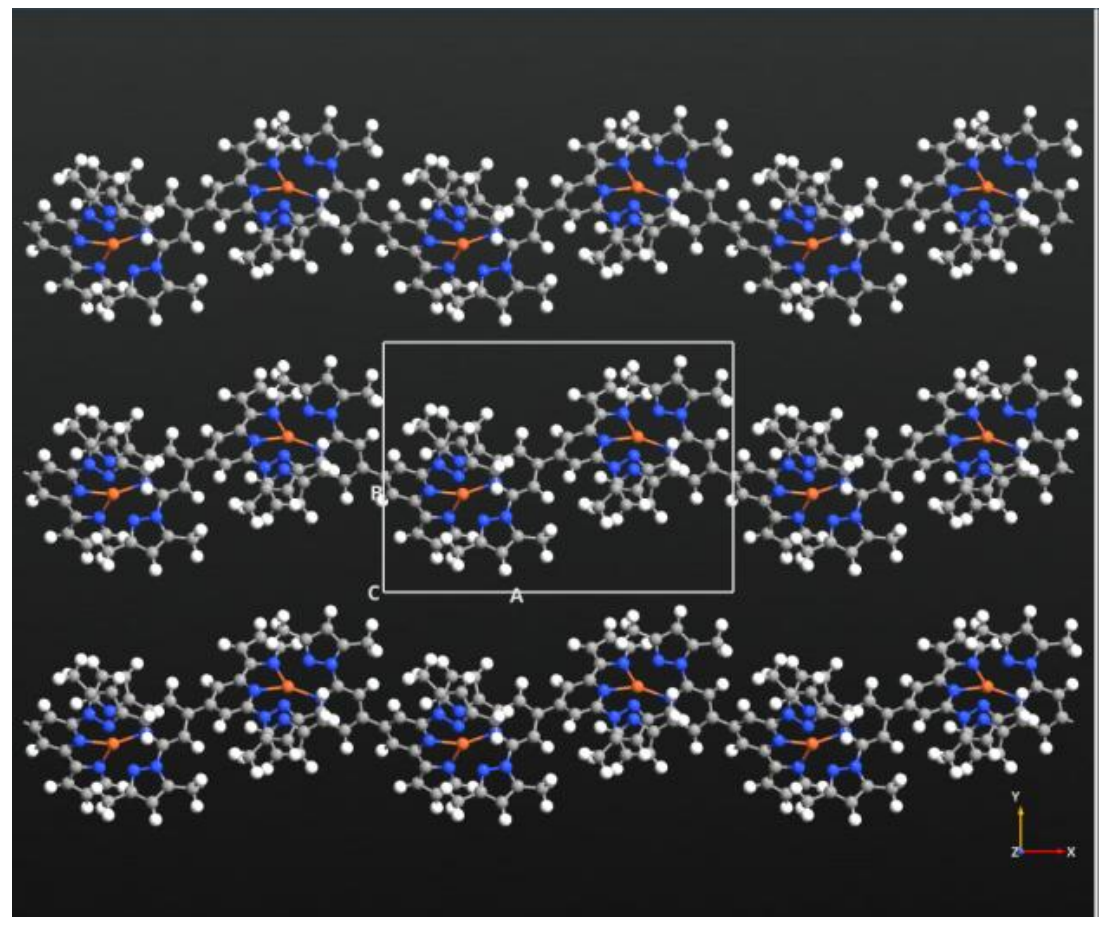

Figure S14: DFT optimized structures of three L-R-L-R segments. The unit cell, denoted by the white rectangle, comprises two molecules with a lattice vector of 2.1 nm.

6. Analytical Data

^{1}H-NMR and ^{13}C-NMR spectra

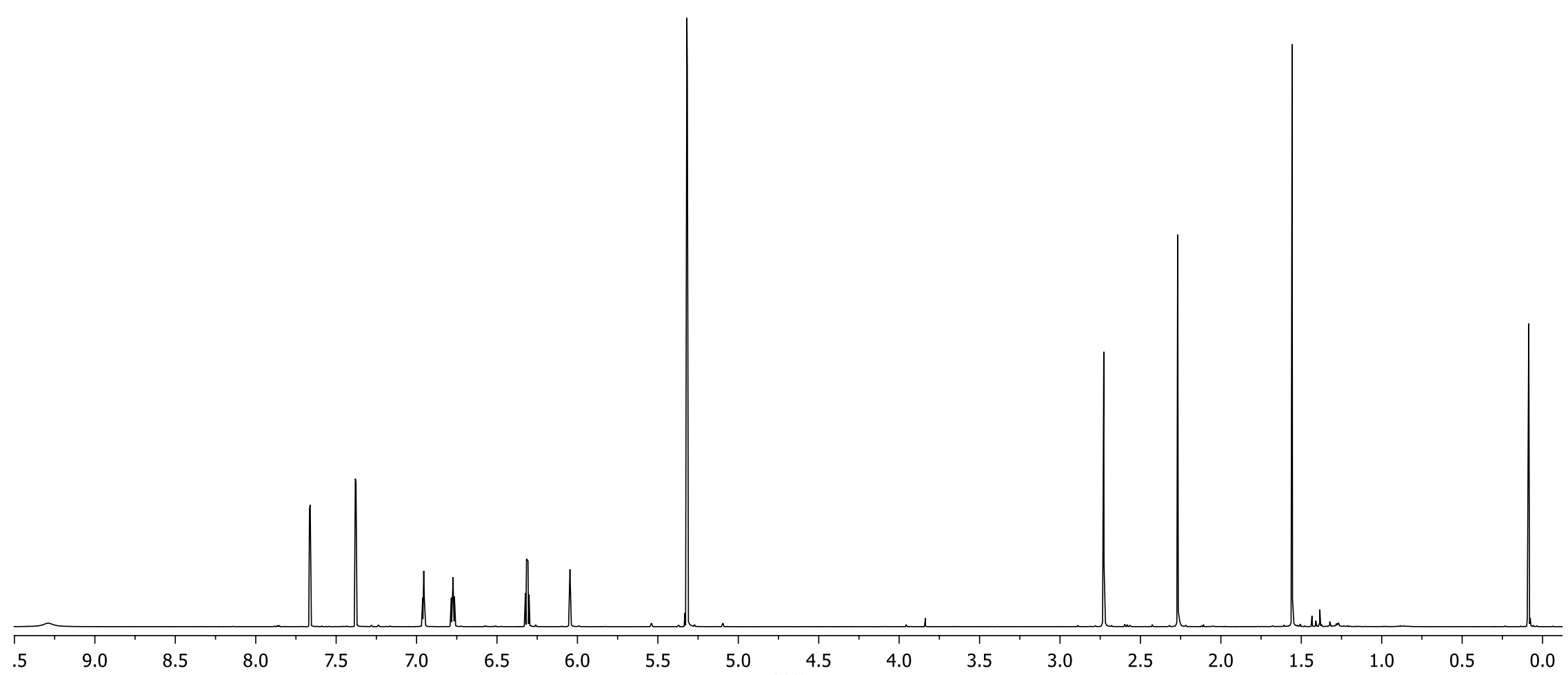

Figure S15: **4-Chloro-2-(3,5-dimethyl-1H-pyrazol-1-yl)-6-(1H-pyrrol-2-yl)pyridine (pzpypyr-Cl)** ^{1}H-NMR spectrum.

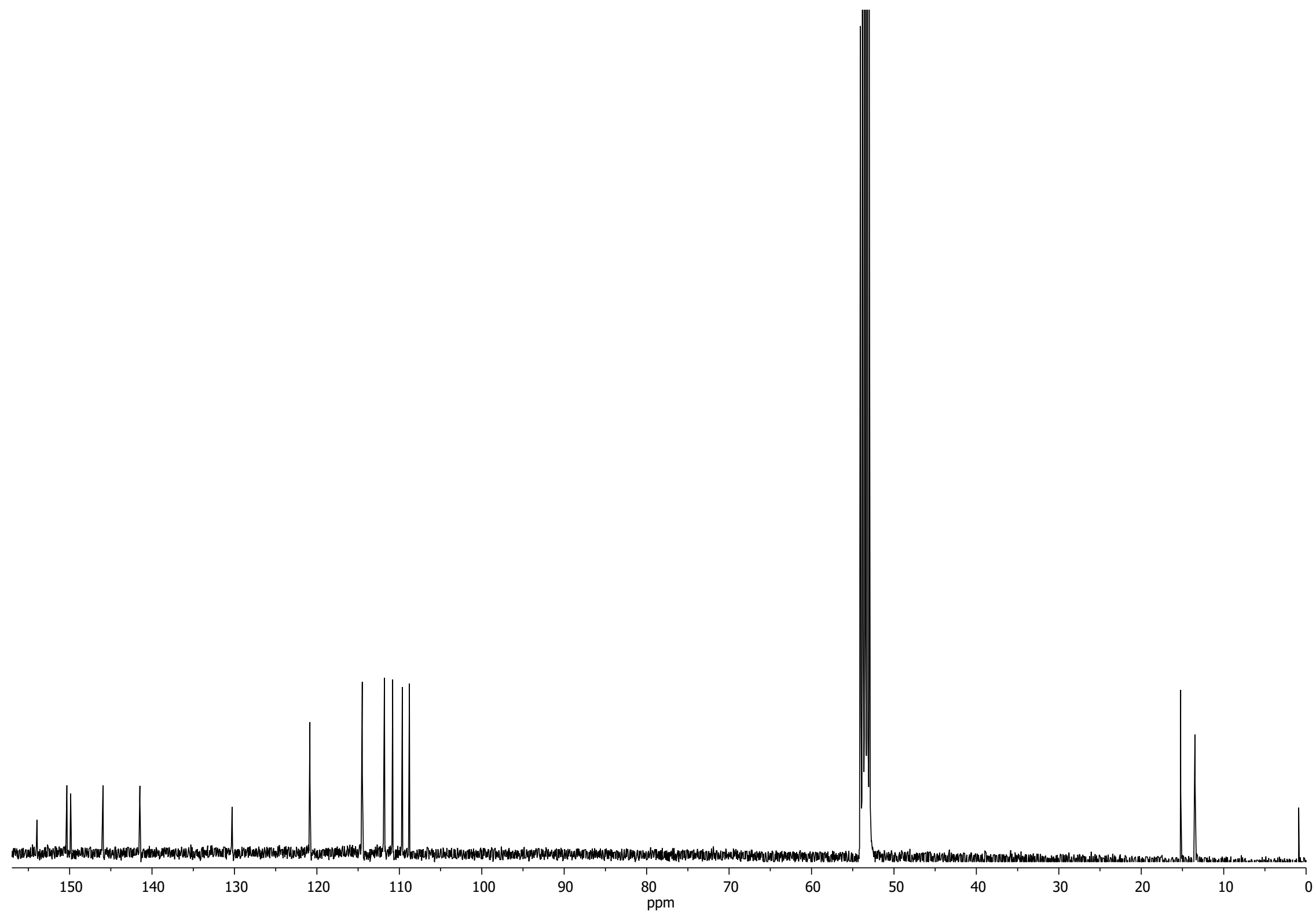

Figure S16: **4-Chloro-2-(3,5-dimethyl-1H-pyrazol-1-yl)-6-(1H-pyrrol-2-yl)pyridine (pzpypyr-Cl)** ^{13}C-NMR spectrum.

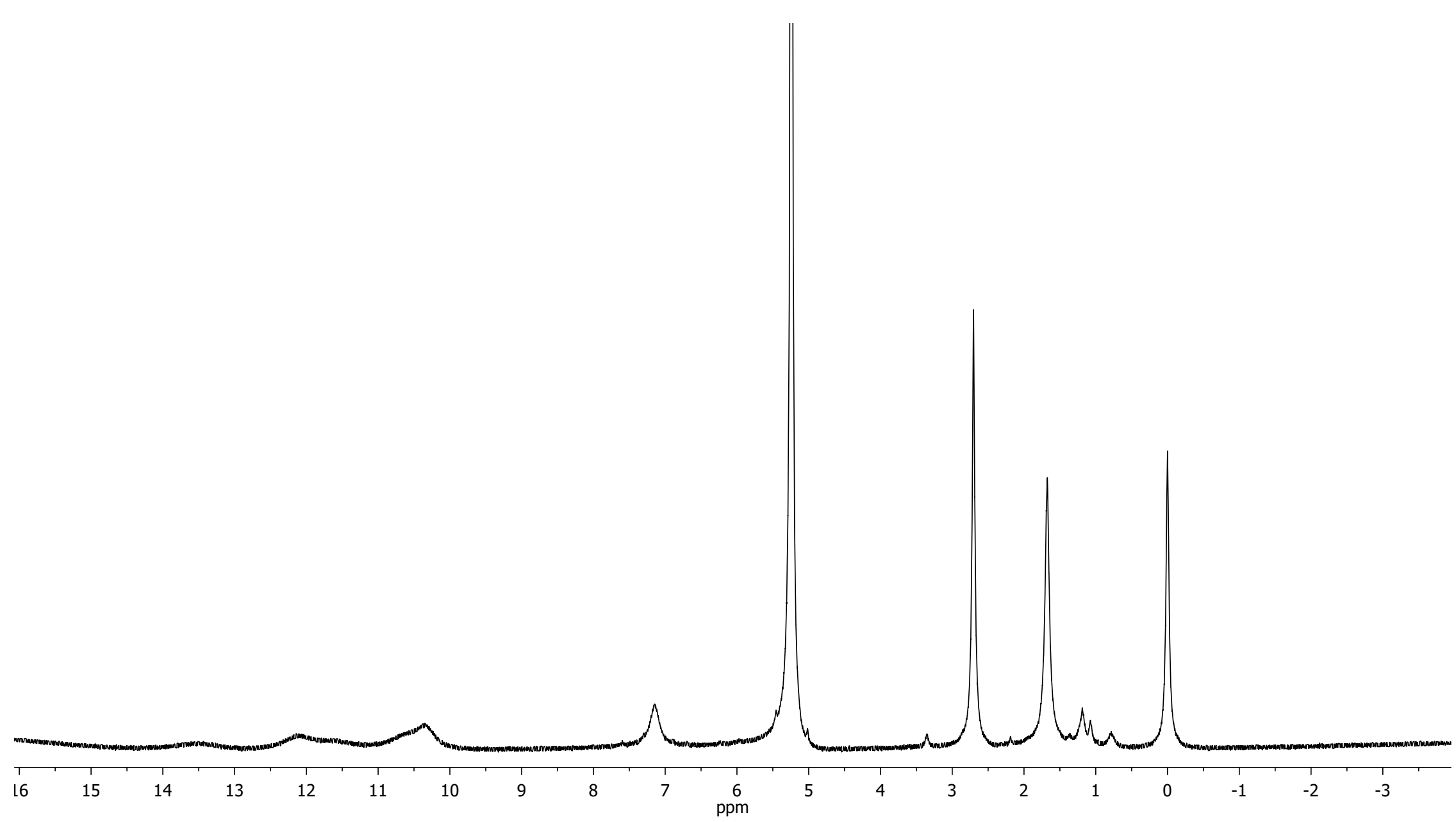

Figure S17: [**Fe(pzpypyr-Cl)**$_2$] ^{1}H-NMR spectrum.

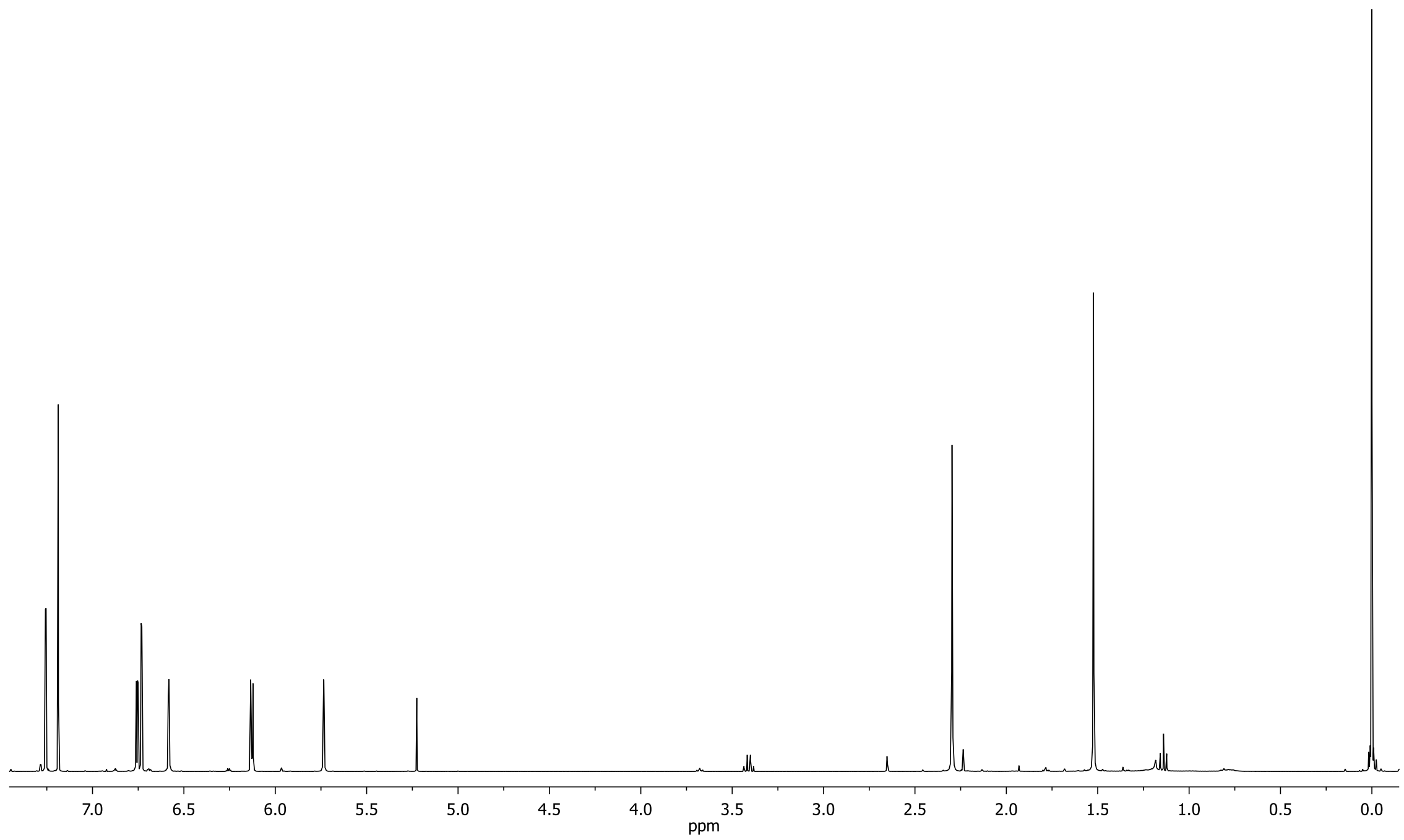

Figure S18: [**Zn(pzpypyr-Cl)**$_2$] ^{1}H-NMR spectrum.

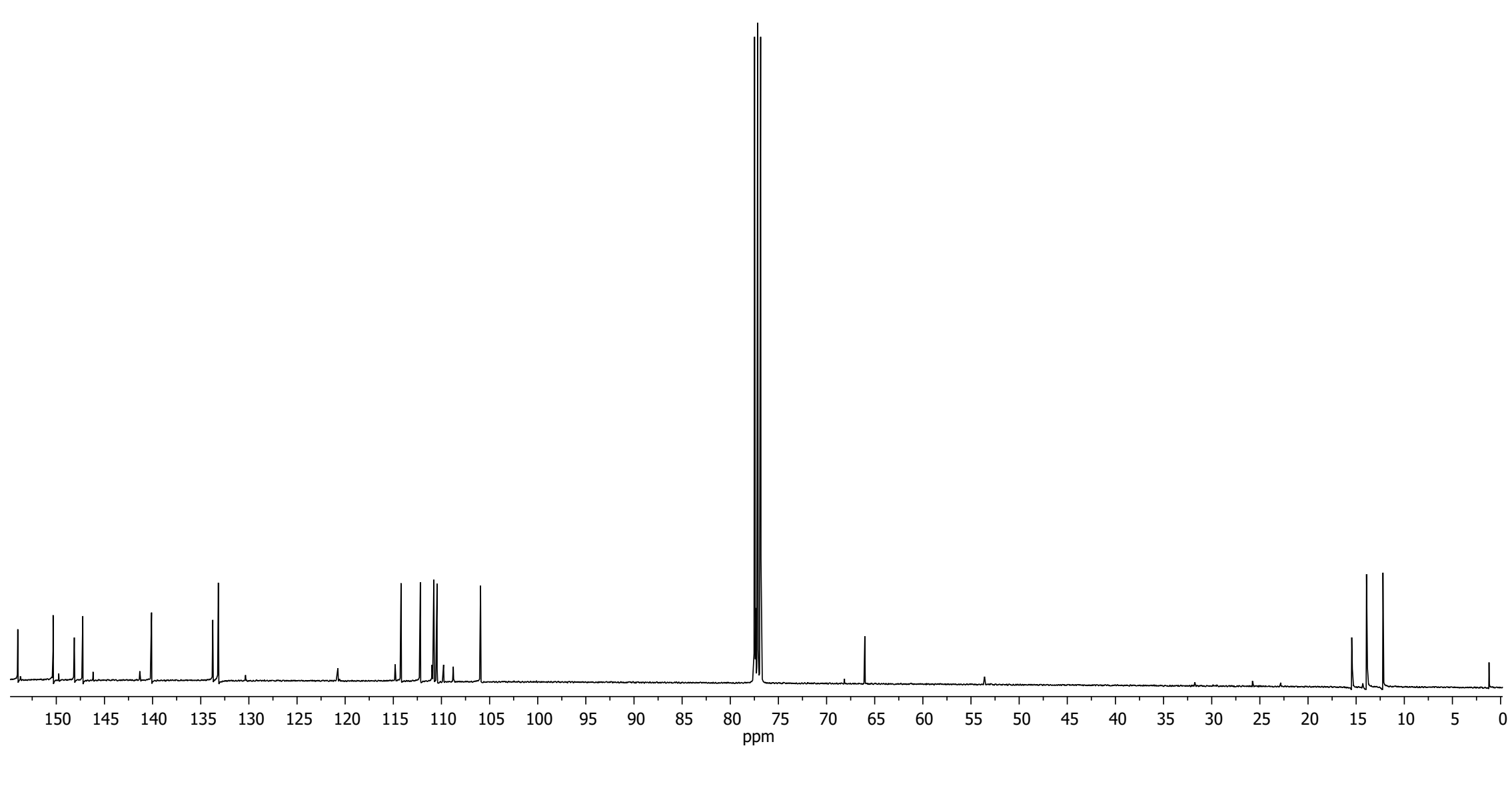

Figure S19: [**Zn(pzpypyr-Cl)**$_2$] ^{13}C-NMR spectrum.

Mass Spectra

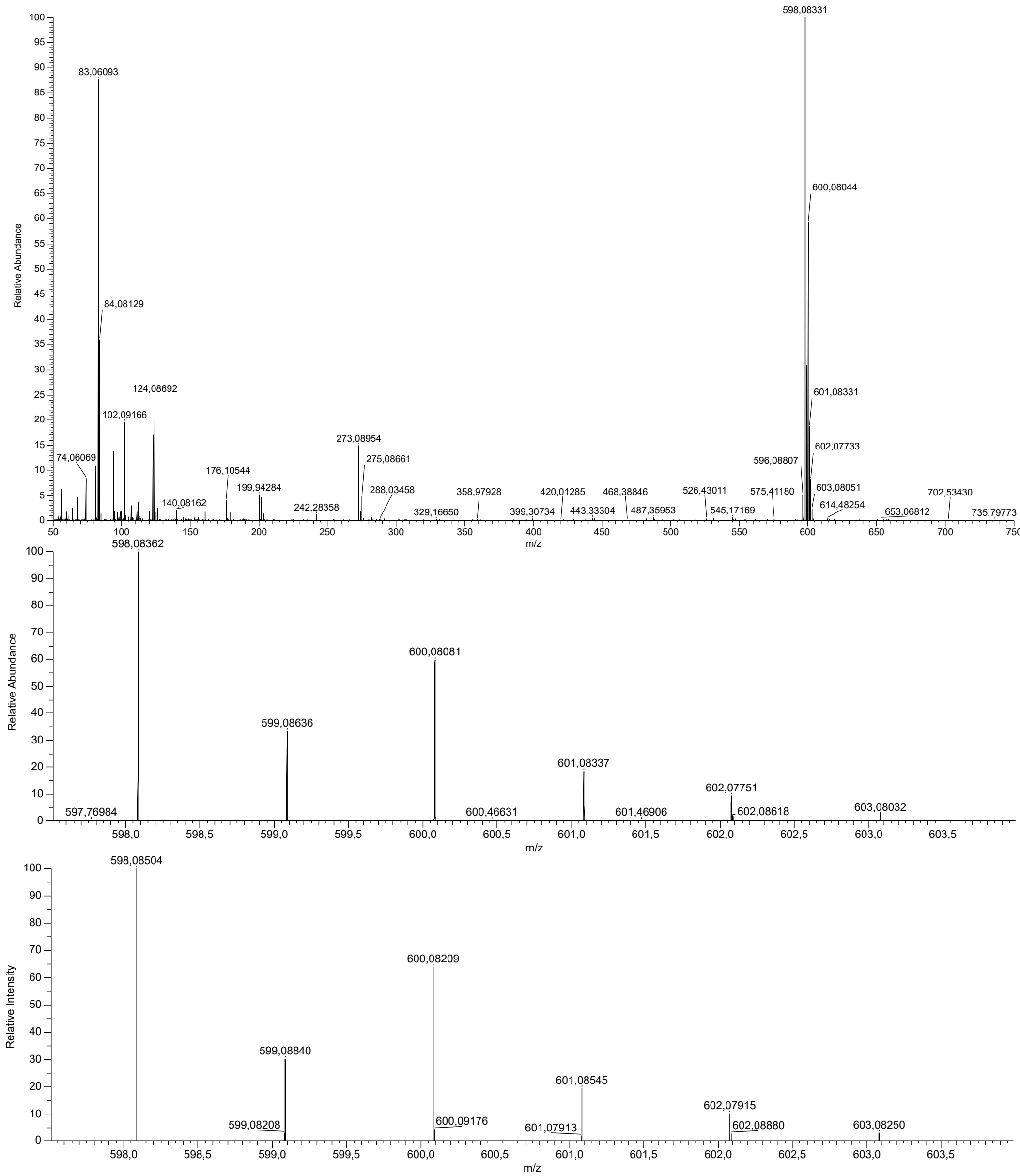

Figure S20: High Resolution ESI-MS spectra of [Fe(pzpypyr-Cl)$_2$]. Top: Measured range; Middle: enlargement of the isotope pattern of [Fe(pzpypyr-Cl)$_2$]; Bottom: calculated mass spectra of [Fe(pzpypyr-Cl)$_2$].

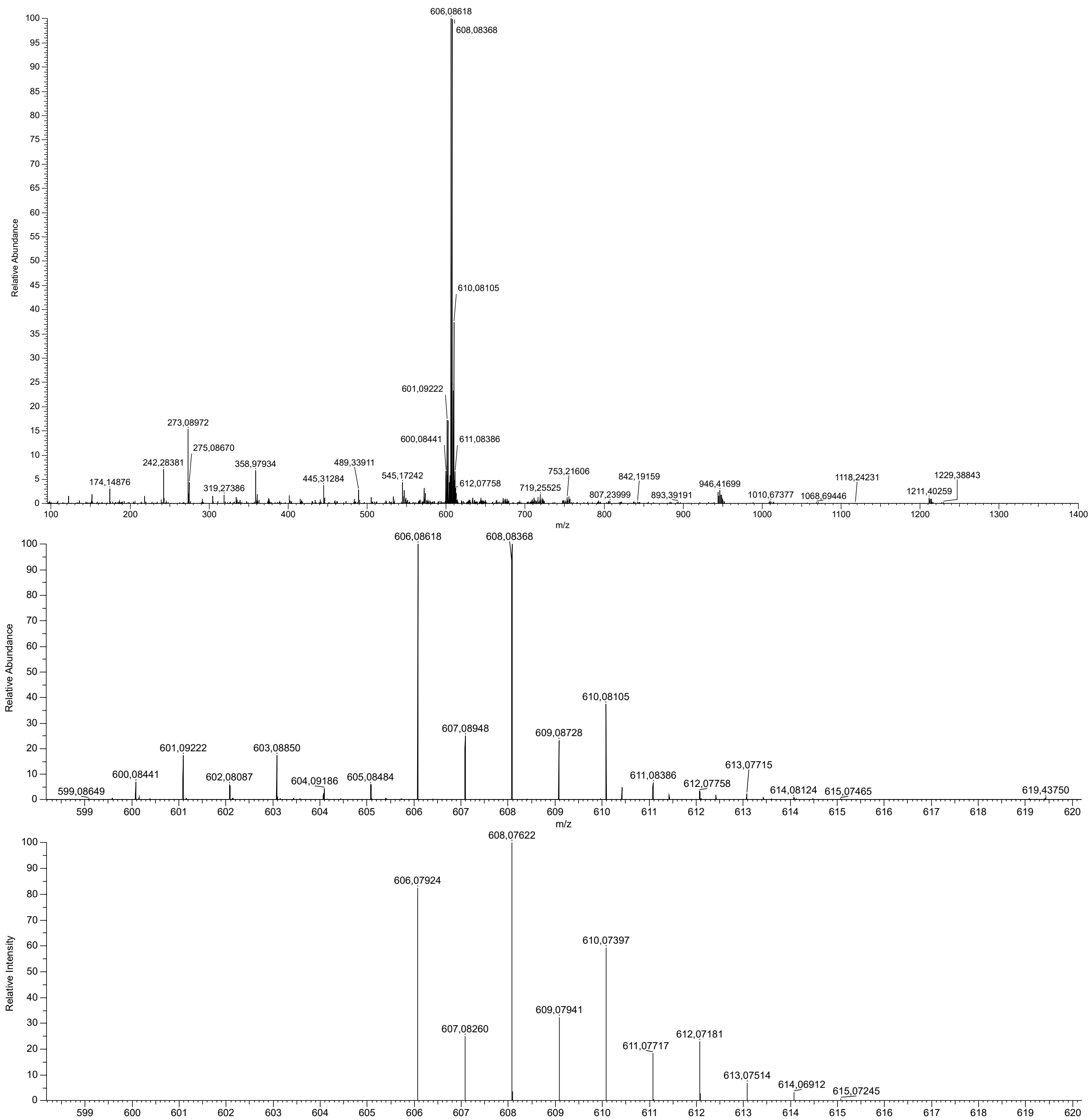

Figure S21: High Resolution ESI-MS spectra of [Zn(pzpypyr-Cl)$_2$]. Top: Measured range; Middle: enlargement of the isotope pattern of [Zn(pzpypyr-Cl)$_2$]; Bottom: calculated mass spectra of [Fe(pzpypyr-Cl)$_2$].

References

1. Smidstrup, S.; Markussen, T.; Vancraeyveld, P.; Wellendorff, J.; Schneider, J.; Gunst, T.; Verstichel, B.; Stradi, D.; Khomyakov, P. A.; Vej-Hansen, U. G.; Lee, M.-E.; Chill, S. T.; Rasmussen, F.; Penazzi, G.; Corsetti, F.; Ojanperä, A.; Jensen, K.; Palsgaard, M. L. N.; Martinez, U.; Blom, A.; Brandbyge, M.; Stokbro, K. QuantumATK: An Integrated Platform of Electronic and Atomic-Scale Modelling Tools. *J. Phys. Condens. Matter* **2020**, *32*, 015901.

2. Perdew, J. P.; Burke, K.; Ernzerhof, M. Generalized Gradient Approximation Made Simple. *Phys. Rev. Lett.* **1996**, *77*, 3865.

3. Grimme, S.; Antony, J.; Ehrlich, S.; Krieg, H. A Consistent and Accurate *ab Initio* Parametrization of Density Functional Dispersion Correction (DFT-D) for the 94 Elements H-Pu. *J. Chem. Phys.* **2010**, *132*, 154104.

4. Jameson, D. L.; Goldsby, K. A. 2,6-bis(*N*-pyrazolyl)pyridines: the Convenient Synthesis of a Family of Planar Tridentate N_3 Ligands that are Terpyridine Analogs. *J. Org. Chem.* **1990**, *55*, 4992–4994.

5. Luconi, L.; Gafurov, Z.; Rossin, A.; Tuci, G.; Sinyashin, O.; Yakhvarov, D.; Giambastiani, G. Palladium (II) Pyrazolyl–Pyridyl Complexes Containing a Sterically Hindered N-Heterocyclic Carbene Moiety for the Suzuki-Miyaura Cross-Coupling Reaction. *Inorg. Chim. Acta* **2018**, *470*, 100–105.

6. Grunwald, J.; Torrès, J.; Buchholz, A.; Näther, C.; Kämmerer, L.; Gruber, M.; Rohlf, S.; Thakur, S.; Wende, H.; Plass, W.; Kuch, W.; Tuczek, F. Defying the Inverse Energy Gap Law: a Vacuum-Evaporable Fe (II) Low-Spin Complex with a Long-Lived LIESST State. *Chem. Sci.* **2023**, *14*, 7361–7380.

7. Böttger, M.; Wiegmann, B.; Schaumburg, S.; Jones, P. G.; Kowalsky, W.; Johannes, H.-

H. Synthesis of New Pyrrole–Pyridine-Based Ligands Using an in Situ Suzuki Coupling Method. *Beilstein J. Org. Chem.* **2012**, *8*, 1037–1047.
